# Enzyme-responsive progelator cyclic peptides for minimally invasive delivery to the heart post-myocardial infarction

**DOI:** 10.1038/s41467-019-09587-y

**Published:** 2019-04-15

**Authors:** Andrea S. Carlini, Roberto Gaetani, Rebecca L. Braden, Colin Luo, Karen L. Christman, Nathan C. Gianneschi

**Affiliations:** 1 Department of Chemistry & Biochemistry, University of California, San Diego, La Jolla, CA 92093 USA; 2 Department of Chemistry, Department of Materials Science & Engineering, Department of Biomedical Engineering, Simpson Querrey Institute for BioNanotechnology, International Institute for Nanotechnology, and Chemistry of Life Processes Institute, Northwestern University, Evanston, IL 60208 USA; 3 Department of Bioengineering, Sanford Consortium for Regenerative Medicine, University of California, San Diego, La Jolla, CA 92093 USA

## Abstract

Injectable biopolymer hydrogels have gained attention for use as scaffolds to promote cardiac function and prevent negative left ventricular (LV) remodeling post-myocardial infarction (MI). However, most hydrogels tested in preclinical studies are not candidates for minimally invasive catheter delivery due to excess material viscosity, rapid gelation times, and/or concerns regarding hemocompatibility and potential for embolism. We describe a platform technology for progelator materials formulated as sterically constrained cyclic peptides which flow freely for low resistance injection, and rapidly assemble into hydrogels when linearized by disease-associated enzymes. Their utility in vivo is demonstrated by their ability to flow through a syringe and gel at the site of MI in rat models. Additionally, synthetic functionalization enables these materials to flow through a cardiac injection catheter without clogging, without compromising hemocompatibility or cytotoxicity. These studies set the stage for the development of structurally dynamic biomaterials for therapeutic hydrogel delivery to the MI.

## Abstract (web summary)

Injectable hydrogels have gained significant interest; yet, due to high viscosity, many are unsuitable for catheter delivery. Here, the authors report on cyclic peptides with low viscosity for catheter delivery, which form self-assembled peptide hydrogels following enzymatic cleavage and demonstrated delivery in vivo.

## Introduction

Myocardial infarction (MI) accounts for 46% of all deaths attributed to cardiovascular disease^1^. Within the first few days post MI, an inflammatory response causes cardiomyocyte death and degradation of the native extracellular matrix (ECM) by secreted matrix metalloproteinases (MMPs)^2^. This in turn can lead to aneurysmal thinning and negative left ventricular (LV) remodeling within days to weeks. Left untreated, heart failure results as one of the leading causes of death in the western world.

Injectable hydrogel-based scaffolds have gained attention as a therapeutic approach to prevent negative LV remodeling by utilizing materials to stimulate cardiac repair. Materials for this purpose have included myocardial ECM^3^, alginate^4^, and hyaluronic acid hydrogels^5^, or employing biomaterials as therapeutic delivery scaffolds such as naturally derived polymeric hydrogels (e.g., collagen^6^, fibrin^7^, heparin^8^, and gelatin^9^), synthetic polymeric hydrogels (e.g., poly(*N*-isopropylacrylamide) (PNIPAAm)^10^, ureido-pyrimidinone-modified poly(ethylene glycol) (PEG)^11^, and chitosan^12^), and microparticles (e.g., poly (lactic-co-glycolic acid) (PLGA)^13^ and dextran^14^). Despite many successful preclinical studies, wide spread translation and initiation of clinical trials has been slow, with current studies limited to a myocardial ECM hydrogel (clinicaltrials.gov identifier NCT02305602) and alginate (NCT3082508 and NCT01311791). One reason for this lack of translation is that the majority of these hydrogels are not candidates for minimally invasive catheter delivery because of excess material viscosity, their quick gelling nature that can lead to catheter clogging, and concerns regarding hemocompatibility since materials can leak into the bloodstream upon injection into a beating heart^15–17^. One versatile class of materials that has been successfully tested in several preclinical MI models using surgical epicardial injections is self-assembling peptides (SAPs)^18–21^. SAP hydrogels are attractive as they: (1) resemble native ECM, (2) require no additive reagents to induce gelation, (3) are biodegradable, (4) are biocompatible, (5) have pore sizes (~5–200 nm) conducive to promote endothelial cell adhesion and capillary formation, (6) allow rapid cellular migration because of their flexibility, (7) are rehealable, (8) do not suffer from batch-to-batch chemical variability, and (9) are amenable to sequence modification^22,23^. However, SAPs have not demonstrated amenability to cardiac catheter delivery.

Previously, we demonstrated that soluble peptide–polymer-based nanoparticles can aggregate into macromolecular scaffolds in diseased tissue through the action of endogenously expressed inflammatory-related enzymes, MMPs, providing a viable method for targeted accumulation^24^, prolonged tissue retention^25^, and therapeutic delivery^26^. However, these materials employed a non-biodegradable functionalized polynorbornene backbone and did not possess viscoelastic properties. Inspired by recent efforts with enzyme-responsive peptides^27–29^ and a study by Nilsson and coworkers^30^ utilizing reductively triggered cyclic peptides as progelators, we focused on developing a cyclic, enzyme-triggered, responsive peptide platform that would enable minimally invasive delivery of SAPs to the heart. Specifically, MMP-2/9^2,31^ and elastase^32,33^, which are endogenously expressed at the site of MI during the acute inflammatory (days) and fibrotic (weeks) phases, provide a useful handle, playing key roles in the healing process through degradation of ECM and fibrinogen^2^.

**Fig. 1:**
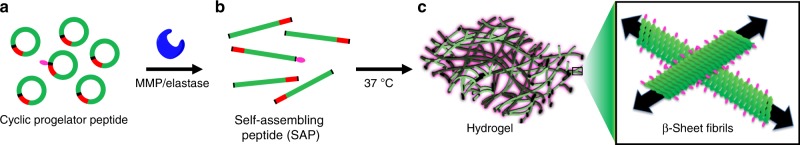
Design of cyclic, enzyme-responsive progelator peptides for activatable gelation. **a** Cyclic progelator peptides, containing gelling sequence (green), matrix metalloproteinase (MMP)/elastase enzyme cleavage recognition sequence (red), and disulfide bridge (black), resist assembly due to conformational constraint. Rhodamine-labeled (pink ellipse) self-assembling peptides (SAPs) were employed for in vivo studies as a 5 mol% additive to provide a means for imaging the hydrogels in ex vivo microscopy analyses. **b** Enzymatic cleavage results in linearization into SAPs. **c** SAPs assemble into viscoelastic hydrogels composed of β-sheets fibrils

In this work, we engineer cyclic peptide progelators that flow freely in solution until proteolytically activated (Fig. 1). Model SAP sequences are prepared as water-soluble, dispersed cyclic progelators (green rings, Fig. 1a) that contain a substrate recognition sequence for MMP-2/9 and elastase (red) and a handle for fluorescent labeling. We show that enzymatic cleavage of these sterically constrained cyclic progelators results in linearization to generate SAPs (Fig. 1b) which subsequently assemble into rehealable viscoelastic hydrogels (Fig. 1c). Their low viscosity, ability to gel at the site of MI, and hemocompatible nature of the concentrated progelator are demonstrated. In addition, we show that the progelators are amenable to minimally invasive catheter injection using an in vitro model system. This opens the possibility of delivering SAPs to the heart via catheter.

## Results

### Design of responsive SAPs for activatable gelation in vivo

**Fig. 2:**
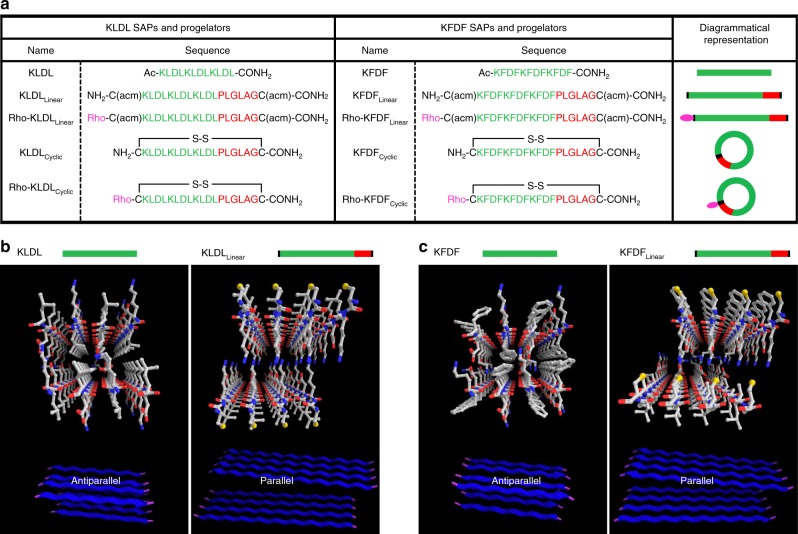
Sequences and design of self-assembling peptides (SAPs) and cyclic, enzyme-responsive progelators. **a** Table of SAPs and progelators based on the (KLDL)_3_ and (KFDF)_3_ amino acid gelling sequence with corresponding diagrammatical representations (green is progelator sequence, red is recognition sequence, pink is fluorophore). Sequences are shown for the base, unmodified SAPs (KLDL and KFDF) and modified SAPs (KLDL_Linear_ and KFDF_Linear_) primed for cyclization but lacking fluorescent labels, together with the corresponding cyclized versions (progelators, KLDL_Cyclic_ and KFDF_Cyclic_). In addition, rhodamine-labeled modified SAPs (Rho-KLDL_Linear_ and Rho-KFDF_Linear_) are shown, together with their corresponding labeled cyclized progelators (Rho-KLDL_Cyclic_ and Rho-KFDF_Cyclic_). The ‘acm' denotes the acetamidomethyl protecting group on Cysteine residues. ‘Rho' designates the 5(6)-carboxytetramethyl rhodamine label. **b**, **c** Modeling predicts β-sheet re-orientation, rather than self-assembly disruption, following significant SAP functionalization. **b** Predicted structures and ribbon diagrams (bottom) of KLDL and KLDL_Linear_ SAPs from FibPredictor simulations revealing antiparallel and parallel β-sheet orientations, respectively. **c** Predicted structures and ribbon diagrams (bottom) of KFDF and KFDF_Linear_ SAPs from FibPredictor simulations revealing antiparallel and parallel β-sheet orientations, respectively (see Supplementary Tables 1-2). Ball and stick models of interacting peptides are displayed with carbon, oxygen, and nitrogen atoms in white, red, and blue, respectively

SAPs undergo spontaneous assembly through electrostatic and amphiphilic interactions into ordered nanostructures^34,35^. In many instances, variation of sequence and charge distribution in SAPs can influence properties such as secondary structure^36^, fiber diameter^37^, and bulk viscoelasticity^38^. In our design, we began with a gellable core based on a model SAP consisting of the repeat sequence (KLDL)_3_ (referred to in this study as KLDL), which has been studied as a non-immunogenic, non-hemolytic, and antimicrobial scaffold for tissue engineering applications^39–41^. This peptide self-assembles spontaneously into β-sheets through cationic and anionic crosslinking of the Lys and Asp residues and hydrophobic interactions along the Leu residues. As a proof of concept for sequence control, we chose to employ an analog of KLDL hypothesized to alter secondary structural assembly and achieve modified gel properties. For this purpose, we designed (KFDF)_3_ (referred to as KFDF), in which the incorporation of aromatic Phe residues introduce π–π interactions. From the KLDL and KFDF SAPs, we engineered a small library of functionalized SAPs and progelators for studying assembly within a biological relevant environment (Fig. 2 and Supplementary Figs. 1–3). We first evaluated whether the addition of non-gelling amino acids from the MMP-2/9 recognition sequence (PLG|LAG) and cysteine residues used for macrocyclization into the SAP sequences would interfere with their ability to self-assemble (Fig. 2a). Predictive computational modeling with open-access FibPredictor software^42^ provided initial evidence that functionalized KLDL_Linear_ and KFDF_Linear_ SAPs would experience a switch in self-assembly orientation from antiparallel to parallel β-sheets. Regardless, such a design would retain the capacity to form fibrillar bilayers (Fig. 2b, c and Supplementary Tables 1–2). Notably in KFDF_Linear_, Phe residues on each strand are sandwiched with analogous residues on neighboring strands, which facilitate π–π stacking and stronger hydrophobic interactions than those of Leu-containing peptides. Indeed, with the design validated in silico, synthetic preparation of the KFDF SAP resulted in materials with increased mechanical strength without sacrificing re-healing capacity, inherent to the known KLDL system^43^ (Supplementary Fig. 4).

**Fig. 3:**
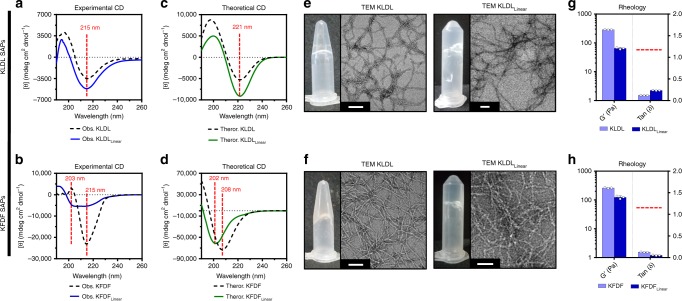
Impact of self-assembling peptide (SAP) functionalization on assembly properties. **a** Experimental circular dichroism (CD) spectra of KLDL (black dashed line) and KLDL_Linear_ (blue line) SAPs, with labeled minimum at 215 nm (n → π*). **b** Experimental CD spectra of KFDF (black dashed line) and KFDF_Linear_ (blue line) SAPs, with labeled minima at 215 nm (n → π*) and 203 nm (π → π*). **c** Theoretical CD spectra of KLDL (black dashed line) and KLDL_Linear_ (green line) SAPs from DichroCalc simulations using FibPredictor models. **d** Theoretical CD spectra of KFDF (black dashed line) and KFDF_Linear_ (green line) SAPs from DichroCalc simulations using FibPredictor models (see Supplementary Tables 3-4). CD spectra minima denoted with dashed lines. **e** Assembled hydrogel photographs and corresponding transmission electron microscopy (TEM) images of the KLDL (left) and KLDL_Linear_ (right) SAPs. **f** Assembled hydrogel photographs and corresponding TEM images of the KFDF (left) and KFDF_Linear_ (right) SAPs. Scale bar 50 nm. **g** Viscoelastic measurements of storage moduli, G’ (Pa), and damping factor, tanδ, for KLDL (light blue) and KLDL_Linear_ (dark blue). **h** Viscoelastic measurements of storage moduli, G’ (Pa), and damping factor, tanδ, for KFDF and KFDF_Linear_. Gel capacity is defined as tanδ < 1 (dashed line). CD measurements performed at 500 µM SAP in 10 mM Tris buffer, pH 7.4 (*n* = 3 repeats). SAPs for TEM and rheology/photographs prepared at 100 µM and 15 mg mL^−1^ in 1× Dulbecco's phosphate-buffered saline (DPBS; pH 7.4), respectively. Rheological measurements reported for angular frequency of 2.5 rad s^−1^ (*n* = 3 repeats). Values are mean ± SEM

In practice, modified SAPs KLDL_Linear_ and KFDF_Linear_, retained the capacity to self-assemble (both experimentally and predicted) despite modest changes in secondary structure and viscoelastic properties (Fig. 3). Experimental circular dichroism (CD) demonstrated that KLDL_Linear_ and KFDF_Linear_ retained β-sheet conformations (n → π* transition at 215 nm). However, the positive peak at ~200 nm (π → π* transition) found in KLDL and KFDF is blue-shifted to ~195 nm, indicative of a parallel to antiparallel orientation switch (Fig. 3a, b)^44^. Additionally, KFDF_Linear_ reveals a new high energy minimum at 203 nm corresponding to π–π* effects resulting from aromatic π–π interactions in the hydrophobic interior of the bilayer structure (Fig. 3b)^30^. This transition is predicted in theoretical spectra from DichroCalc^45^ for KFDF_Linear_ (202 nm) and not for KLDL_Linear_, which contains no aromatic residues (Fig. 3c, d). Regardless of altered secondary structure, fiber morphology and bulk viscoelastic properties exhibited minimal changes to that of unmodified KLDL and KFDF (Fig. 3e–h). Slight drops in storage moduli for KLDL_Linear_ and KFDF_Linear_ are attributed to the decreasing molar concentration of amino acids (hydrogels prepared with respect to weight at 15 mg mL^−1^) contributing to β-sheet formation. Regardless, no significant disruptive effects to gelling capacity, as defined by tanδ < 1 (Fig. 3g, h) or strain tolerance (Supplementary Fig. 5), were observed through the introduction of over 66% more amino acid residues to the SAP sequence. This exemplifies the robustness of these linear SAPs to sequence modification and potential capacity to tolerate functional moieties such as fluorescent tags or small molecule drugs.

### Cyclization and progelator formulation

**Fig. 4:**
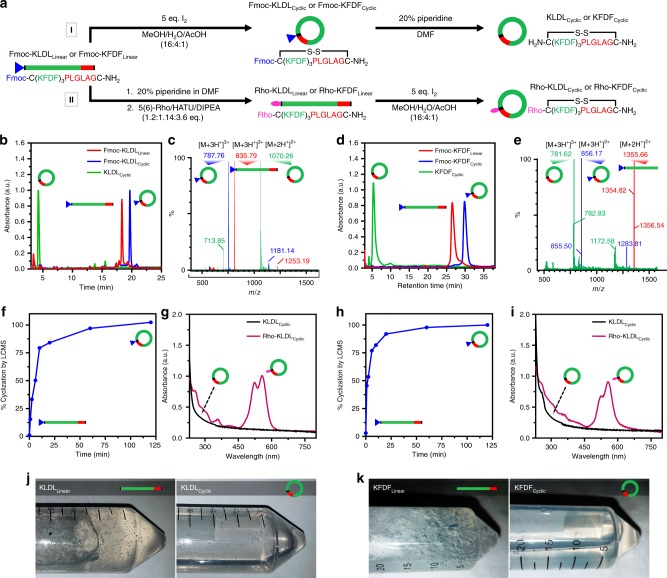
Facile synthesis of sterically constrained and labeled cyclic progelators. **a** Synthetic scheme for unlabeled (route I) and labeled (route II) KLDL_Cyclic_ and KFDF_Cyclic_ progelators. All self-assembling peptides (SAPs) were cyclized under dilute conditions (500 µM) with 5 eq I_2_ in a mixture of AcOH/MeOH/H_2_O to create a soluble progelator. Route I uses N-terminal Fmoc-protected SAPs to improve material separation during chromatographic purification. Fmoc removal generates the intended unlabeled progelators KLDL_Cyclic_ and KFDF_Cyclic_. Route II depicts the synthesis of labeled progelators through N-terminal modification with 5(6)-carboxytetramethyl rhodamine prior to cyclization. **b**, **c** High-performance liquid chromatography (HPLC) (**b**) monitored at 214 nm and corresponding electrospray ionization (ESI) spectra (**c**) of Fmoc-KLDL_Linear_ (red), Fmoc-KLDL_Cyclic_ (blue), and KLDL_Cyclic_ (green) (835.79 *m/z*, 787.76 *m/z*, and 1070.26 *m/z*, respectively) verify purity and mass of synthetic modifications to KLDL peptides. **d**, **e** HPLC (**d**) monitored at 214 nm and corresponding ESI spectra (**e**) of Fmoc-KFDF_Linear_ (red), Fmoc-KFDF_Cyclic_ (blue), and KFDF_Cyclic_ (green) (781.862 *m/z*, 856.17 *m/z*, and 1355.66 *m/z*, respectively) verify purity and mass of synthetic modifications to KFDF peptides. In both KLDL and KFDF systems, macrocyclization causes a slight decrease in polarity and Fmoc-deprotection increases progelator polarity. **f** KLDL_Linear_ SAP macrocyclization kinetics monitored by liquid chromatography–mass spectrometry (LCMS) at 214 nm reveal complete cyclization after 120 min. **g** Absorbance spectra of unlabeled (black) and labeled KLDL progelators (pink, λ_max_ = 565 nm for rhodamine signal). **h** KFDF_Linear_ SAP macrocyclization kinetics monitored by LCMS at 214 nm reveal complete cyclization after 120 min. **i** Absorbance spectra of unlabeled (black) and labeled KFDF progelators (pink, λ_max_ = 565 nm for rhodamine signal). **j** Photographs of KLDL_Linear_ precursor as a hydrogel (left) and the resulting KLDL_Cyclic_ progelator as a soluble solution (right). **k** Photographs of KLDL_Linear_ precursor as a hydrogel (left) and the resulting KLDL_Cyclic_ progelator as a soluble solution (right)

SAP steric constraint provides a simple and versatile engineering approach for preventing network assembly in a biological environment (Fig. 4). Functionalized SAP analogs containing an Fmoc protecting group or rhodamine dye on the N terminus were cyclized through oxidation of terminal cysteine residues to generate unlabeled and labeled progelators (Fig. 4a–e). Dilute solution phase macrocyclization was complete within 125 min by liquid chromatography–mass spectrometry (LCMS) (Fig. 4f, h). Combined, matrix-assisted laser desorption/ionization (MALDI), high‐resolution mass spectrometry (HRMS), and tandem-mass spectrometry (MS) confirm synthesis of the cyclic products (Supplementary Fig. 6–8). Ultraviolet (UV) spectra confirm the presence of rhodamine absorbance in Rho-KLDL_Cyclic_ and Rho-KFDF_Cyclic_ progelators (Fig. 4g, i). Pure nonviscous progelators (Fig. 4j, k) were formulated with a pH switch from basic to neutral conditions into 1× Dulbecco’s phosphate-buffered saline (DPBS), pH 7.4 (10 mM peptide) to minimize aggregation, as determined by light scattering and transmission electron microscopy (TEM; Supplementary Figs. 9–11). Conformational rigidification via macrocyclization is suspected to induce limited oligomerization via intermolecular stacking of cyclic constructs into transient nanotubes (Supplementary Fig. 12), as has been reported in the literature^46,47^. Despite this observed secondary structure, large assemblies were absent by TEM (Supplementary Fig. 10c). Thus, our formulated cyclic progelators persist as free-flowing solutions for easy injection in vivo.

### Recognition of progelators by inflammatory enzymes

**Fig. 5:**
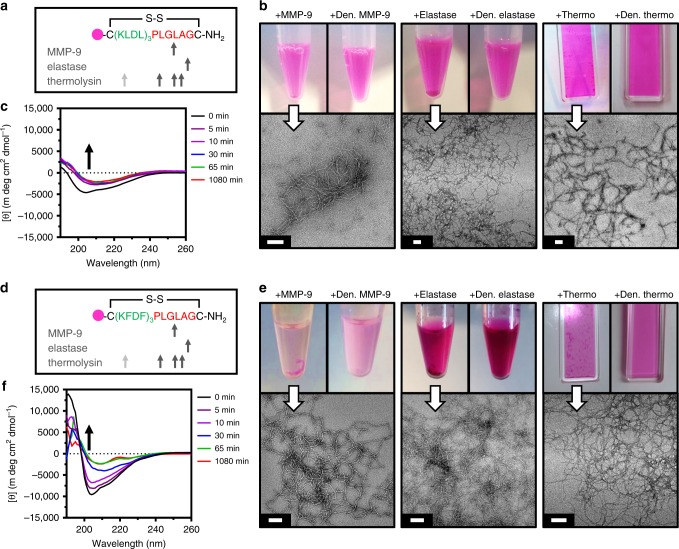
Enzyme responsiveness of Rho-KLDL_Cyclic_ and Rho-KFDF_Cyclic_ progelators. **a**–**c** Analysis of Rho-KLDL_Cyclic_ responsiveness to matrix metalloproteinase-9 (MMP-9), elastase, and thermolysin. **a** Peptide sequence and theoretical enzymatic cuts sites. Gray arrow (not observed). **b** Photographs (top) of progelator incubated with active enzymes (left insets) shows material aggregation and settling from solution, and progelator incubated with denatured enzymes (right insets) show fully dispersed peptide solutions. Corresponding transmission electron microscopy (TEM) images (bottom) of active enzyme cleavage products show fiber formation. Scale 100 nm. **c** Circular dichroism (CD) spectra of cleavage kinetics with thermolysin. Disappearance of signal at 204 nm (black arrow), corresponds to ring-opening. **d–f** Analysis of Rho-KFDF_Cyclic_ to responsiveness MMP-9, elastase, and thermolysin. **d** Peptide sequence and theoretical enzymatic cuts sites. Gray arrow (not observed). **e** Photographs (top) of progelator incubated with active enzymes (left insets) shows material aggregation and settling from solution, and progelator incubated with denatured enzymes (right insets) show fully dispersed peptide solutions. Corresponding TEM images (bottom) of active enzyme cleavage products show fiber formation. Scale 100 nm. **f** CD spectra of cleavage kinetics with thermolysin. Disappearance of signal at 204 nm (black arrow) corresponds to ring opening. CD of 500 µM progelator in 10 mM Tris buffer, pH 7.4. Enzyme cleavages performed at 500 µM progelator with 1:1000, 1:250, and 1:4500 enzyme/substrate molar ratio in 1× cleavage buffers (see Methods). Samples diluted to 100 µM for TEM

Labeled cyclic peptide progelators were tested for their responsiveness to inflammatory-related proteases overexpressed post MI (Fig. 5). The initial inflammatory response is marked by the recruitment of abundant neutrophils, which contribute to the release of proteases such as MMPs and elastase in the first days post MI^31,48^. The fibrotic phase (weeks post MI) is notable for residually high MMP concentrations^2,31^ and elevated sources of elastase from neutrophil extracellular traps, remaining weeks after neutrophil apoptosis ^32,33^.

Rho-KLDL_Cyclic_ and Rho-KFDF_Cyclic_ were incubated with MMP-9 catalytic domain and porcine elastase to cleave at preferential cut sites (Fig. 5a, d). Enzymatic cleavage by active MMP-9 and elastase (Supplementary Fig. 13) induced visible aggregation in reaction vials and entangled fibrous meshes by TEM (Fig. 5b, e), whereas denatured enzymes elicited no response. At the site of MI, the presence of very high MMP and elastase concentrations is expected to induce rapid cleavage, forcing the peptide progelator to assemble and solidify at the target site. However, other inflammation-associated enzymes or constitutively expressed extracellular proteases could play a role in nonspecific degradation. Thus, thermolysin was used to assess general stability of our material to excess proteolysis and subsequent hydrogel disassembly in vivo^49^. Despite the promiscuous activity of thermolysin, incubation with Rho-KLDL_Cyclic_ and Rho-KFDF_Cyclic_ induced fiber formation by TEM (Fig. 5b, e) and structural rearrangement by CD (Fig. 5c, f and Supplementary Fig. 14). Potential intramolecular^50^ and intermolecular^51,52^ steric hindrance from these labeled progelators might explain differences in the CD cleavage kinetics. The similar responsiveness of both unlabeled progelators to inflammatory enzymes and resistance to dissolution by thermolysin demonstrate the versatility of our general platform for conformational control.

### Progelators form viscoelastic and rehealable hydrogels

**Fig. 6:**
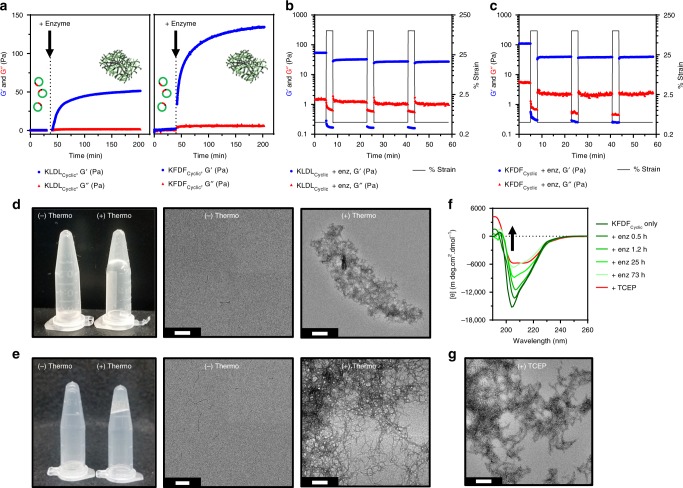
Bulk scale gelation of unlabeled cyclic progelators. **a** Time course rheological analyses of KLDL_Cyclic_ (left) and KFDF_Cyclic_ (right) progelators before and after addition of thermolysin (*t* = ~40 min). Divergence of G’ (blue circles) from G” (red triangles) (G’ > G”) indicates formation of crosslinked hydrogel. Clipart adapted from Servier’s Medical Art database (https://smart.servier.com). **b**, **c** Step-strain oscillation plots of resulting (**b**) linearized KLDL and (**c**) linearized KFDF self-assembling peptide (SAP) hydrogels at 9 h post enzyme activation reveal repeat healing (3 min at 100% strain, 15 min at 0.5% strain, *n* = 3 cycles). Angular frequency 2.5 rad s^−1^, 0.5% strain. **d**, **e** Photographs of (**d**) KLDL_Cyclic_ and (**e**) KFDF_Cyclic_ progelators in the absence (−) and presence (+) of thermolysin with corresponding transmission electron microscopy (TEM). Enzyme cleavages at 10 mM in 1× Dulbecco's phosphate-buffered saline (DPBS) with 1:4500 enzyme/substrate molar ratio. TEM of samples diluted to 100 µM. **f** Circular dichroism (CD) of KFDF_Cyclic_ before and after subsequent additions of thermolysin (shades of green) at 0.5, 1.2, 25, and 73 h shows gradual disappearance of minimum around 204 nm from π–π* interactions between stacked cyclic peptides; 500 µM peptide in 10 mM Tris, pH 7.4. **g**, Tris(2-carboxyethyl)phosphine (TCEP) (red, 1.2 eq)-induced reduction of progelator disulfide bond shows ideal assembly of linearized progelators. Two minima at 204 nm (π → π*) and 215 nm (n → π*) reveal weak aromatic π–π* interactions and β-sheets assembly, respectively; 500 µM peptide in H_2_O, pH 7.4. Scale bars 200 nm

The concept behind our cyclic progelator platform relies on upregulated enzymes to initiate assembly and ultimately viscoelastic gel formation at the site of MI. However, excess proteolysis in vivo from the ‘soup’ of proteases at the MI may prevent gelation or retention of hydrogels at the site of injury. To simulate this environment, we treated our material at 10 mM with a large quantity of the robust and nonspecific enzyme, thermolysin (Fig. 6). Initially, KLDL_Cyclic_ and KFDF_Cyclic_ displayed overlapping storage (G′) and loss (G′′) moduli, indicative of solutions lacking significant crosslinks or chemical interactions (Fig. 6a). Incubation with thermolysin initiates rapid gelation as G′ diverges from G′′. A steady state at 200 min and continuous measurement of viscoelastic properties revealed no change for up to 3 days in the presence of thermolysin. Tightly entangled macromolecular scaffolds are suspected to confer some degree of resistance to further proteolysis. Furthermore, the resulting gels displayed rapid healing capacity when subjected to repeat cycles of excess strain (100%) (Fig. 6b, c). Morphological changes from rheology samples before and after enzyme incubation are illustrated in Fig. 6d, e. Synthetic product analog SAPs in which the gelator sequence contained flanking substrate residues (KLDL_Control_ and KFDF_Control_) were used for simple comparison with enzyme-induced gelation (Supplementary Figs. 15-16). Indeed, secondary assembly characteristics (CD), fibril morphology (TEM), and viscoelastic properties (rheology) of KFDF_Control_ match closely to that of enzymatically cleaved KFDF_Cyclic_ in Fig. 6. We suspect that the slowed kinetics of gel formation for enzyme-treated KLDL_Cyclic_ vs KFDF_Cyclic_ is caused by lower specificity of thermolysin for Leu vs Phe at the P1 position^53^. With hydrogel assembly following close behind, increased proteolytic resistance of tightly packed fibers and limited enzyme diffusion within the porous network^54^ likely has a restrictive effect on further progelator linearization and subsequent gel stiffening for all samples. Regardless, thermolysin cleavage of either progelator generates linear SAPs through the loss of Leu–Gly–Leu (Supplementary Fig. 17).

To understand the initial stages of self-assembly when progelators first encounter their target protease in vivo, slow step-wise cleavage of KFDF_Cyclic_ progelator was achieved through serial additions of dilute thermolysin (Fig. 6f). CD reveals that linearization by enzyme cleavage causes an increase in peptide flexibility, shown by a steep decrease in the signal at 204 nm, and unchanged β-sheet signal from lateral alignment at 215 nm. Tris(2-carboxyethyl)phosphine (TCEP) was used as a positive control to represent ideal assemblies of linearized peptides through complete reduction of the disulfide bond (Fig. 6f and Supplementary Fig. 18). By TEM, reduced peptides assemble as tangled fibrils, similarly to enzymatically cleaved peptides (Fig. 6g). Thus, the enzyme-induced gels observed in Fig. 6 are likely forming through the same self-assembly mechanisms predicted with KFDF_Control_. The rheological and spectroscopic results presented here indicate that our system has potential to tolerate excess proteolytic degradation in vivo.

### Catheter compatibility of cyclic peptide progelators

**Fig. 7:**
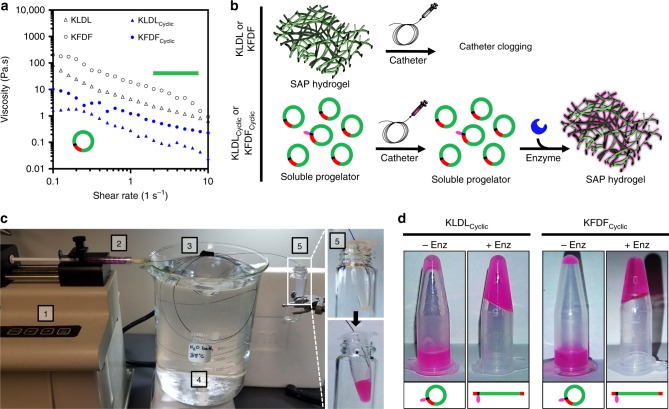
In vitro catheter injection of low-viscosity progelators. **a** Complex viscosity of KLDL_Cyclic_ (blue triangles) and KFDF_Cyclic_ (blue circles) progelators reveal >20× decrease in comparison to corresponding unmodified KLDL (black triangles) and KFDF (black circles) self-assembling peptides (SAPs). **b** Schematic illustrating catheter applicability of KLDL and KFDF SAPs vs that of enzyme-responsive KLDL_Cyclic_ and KFDF_Cylic_ progelators. Injection of SAP hydrogels causes clogging. Clipart adapted from Servier’s Medical Art database (https://smart.servier.com). **c** Experimental setup of progelator (2 mol% labeled) catheter injection test. Peptide was (1) loaded in a 1 mL syringe, (2) mounted on a syringe pump, and (3) flowed at 0.6 mL min^−1^ through the inner nitinol tubing (27 G) of a MyoStar transendocardial injection catheter. The catheter was (4) submerged in a circulating water bath heated to 37 °C. The collection tube (5) before (top) and after (bottom) injection shows successful injection. **d** Photographs of KLDL_Cyclic_ and KFDF_Cyclic_ progelators (2 mol% labeled) after catheter injection into an empty vial (left) or a vial containing thermolysin (right), demonstrating that injection does not disrupt enzyme responsiveness. Progelator formulated as 10 mM in 1× Dulbecco's phosphate-buffered saline (DPBS; pH 7.4) and treated with 1:4500 enzyme/substrate molar ratio

Minimally invasive injection of hydrogels into the heart is typically performed through transendocardial injections. Unlike direct epicardial injections performed in small animal models, transendocardial delivery requires that material should be able to reside within the catheter for up to hour-long procedures, yet still flow over multiple injections during this time, and finally only form a solid gel once it has entered the tissue^17,55^. Rapid gelation and/or high viscosity prohibit many injectable hydrogels from being considered for this minimally invasive delivery route. KLDL_Cyclic_ and KFDF_Cyclic_ progelators were amenable to cardiac catheter injection (Fig. 7). As shown in Fig. 7a, both unmodified SAPs and cyclic progelators exhibit shear-thinning behavior, but the viscosities for the latter are over 20× lower for both KLDL_Cyclic_ and KFDF_Cyclic_. Thus, conformational constraint of β-sheet forming SAPs was sufficient to disrupt favorable unimer alignment and weakened structural interactions. We reasoned that SAP cyclization would be sufficient to prevent clogging during catheter injection (Fig. 7b).

To mimic cardiac catheter injection in vivo, both cyclic progelators (2 mol% labeled for visualization) were flowed through the inner nitinol tubing of a 27 G MyoStar transendocardial injection catheter (Fig. 7c). No excess resistance was detected by manual operation following incubation in the catheter loop for 60 min for either peptide. Complex viscosity of cyclic progelators was independent of peptide concentration up to 10 mM (Supplementary Fig. 19). In contrast, unmodified KLDL and KFDF SAPs caused immediate catheter clogging. Excess resistance from high viscosity and tendency to rapidly self-assemble during shearing injection could explain why there are no previous reports of SAPs for cardiac catheter delivery. To ensure that the shear forces upon cyclic progelators during injection do not inhibit enzyme activation, peptides were injected through the catheter into a vial containing thermolysin and incubated for 3 h (37 °C), resulting in hydrogel formation (Fig. 7e and Supplementary Movie 1).

### Biocompatibility and injection in a rat MI model

**Fig. 8:**
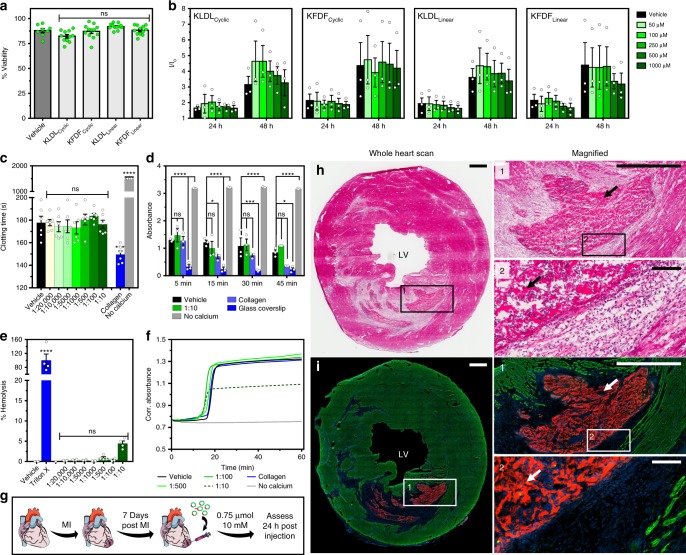
Cell viability, hemocompatibility, and in vivo analysis. **a** Acute and **b** prolonged cytotoxicity of KLDL_Cyclic_, KFDF_Cyclic_, KLDL_Linear_, and KFDF_Linear_ in human cardiac progenitor cells (CPCs). **a** Percent cell viability after peptide (10 mM) incubation for 4 h (*n* = 8–12 repeats in duplicate). **b** Fold increase (*I*/*I*_o_) over baseline of CPC viability after incubation for 24 and 48 h with 0, 50, 100, 250, 500, and 1000 µM peptide (*n* = 3 repeats in quadruplicate). **c**–**f** Hemocompatibility of progelator (shades of green, 1:20,000, 1:10,000, 1:5000, 1:1000, 1:500, 1:100, and 1:10 blood volume dilution of injected dosage) in human blood with positive (collagen, glass coverslip, and 1% Triton X-100), negative (no calcium), and vehicle (1× Dulbecco's phosphate-buffered saline (DPBS)) controls (blue, gray, and black, respectively). **c** Activated clotting times (ACT). No calcium controls are >1500 s (*n* = 6 per group). **d** Whole blood hemostasis kinetics at 5, 15, 30, and 45 min (n = 3 per group). **e** Hemolysis of red blood cells (RBCs) after 1 h of incubation (*n* = 4 per group). **f** Pro-thrombotic profiles in platelet-poor plasma (PPP). Onset of coagulation is accompanied by an increase in absorbance (*n* = 6 per group). **g** In vivo study timeline. Female Sprague-Dawley rats received ischemic reperfusion surgery (35 min occlusion), then a single 75 µL intramyocardial injection (10 mM peptide in 1× DPBS, pH 7.4) of KFDF_Cyclic_ (5 mol% Rho-KFDF_Cyclic_) at 7 days post myocardial infarction (MI) to simulate a local injection. Animals were euthanized at 24 h post injection, and hearts were collected (*n* = 5 animals). Clipart adapted from Servier’s Medical Art database (https://smart.servier.com). **h** Hematoxylin and eosin (H&E)-stained representative heart section. Inset images illustrate hydrogel assembly in the infarct. Arrows show peptide material. **i** Corresponding fluorescence images of the neighboring section, stained for nuclei (blue) and α-actinin (green), with rhodamine-labeled peptide gels in red. LV left ventricle. Scale bars are 1 mm (**h**, **i**, inset 1) and 100 µm (inset 2), respectively. ns (*p* > 0.05), *p ≤ 0.05, ***p ≤ 0.001, and ****p ≤ 0.0001. Ordinary one-way analysis of variance (ANOVA) (**c**, **e**) and two-way ANOVA (**d**) for comparison with vehicle standard. Values are mean ± SEM

In vitro and in vivo tests were conducted to assess practical application using SAP cyclic progelators (Fig. 8). Cytotoxicity of both progelators (KLDL_Cyclic_ and KFDF_Cyclic_) and linear SAPs (KLDL_Linear_ and KFDF_Linear_) were studied with human cardiac progenitor cells (CPCs) (Fig. 8a, b). Exposure at high concentration (10 mM) for 4 h showed no difference in toxicity for all peptides (Fig. 8a). Exposure at 24 and 48 h showed an increase in metabolic activity for all peptides at each concentration and timepoint comparable to growth media only (Fig. 8b). This suggests that our various peptide modifications were not only cytocompatible but also did not influence cell metabolism and proliferation. We next examined the KFDF_Cyclic_ progelator in hemocompatibility studies and assessed in vivo gelation (Fig. 8c–i). Biomaterial leakage is a concern with transendocardial injection since injections are performed into a beating heart^56^. Therefore, we assessed hemocompatibility of the cyclic progelator to ensure that modification and cyclization of the SAPs would not generate a thrombogenic response. Whole human blood clotting times, hemostasis kinetics, red blood cell (RBC) hemolysis, and pro-thrombotic profiles were evaluated in the presence of increasing cyclic peptide progelator concentrations in blood. Activated clotting times (ACT) were used as a standard method^57^ that encompasses intrinsic and common coagulation pathways to assess thrombogenicity. Furthermore, as this method is influenced by increased sample viscosity due to clot formation, potential peptide cleavage by blood proteases and resulting self-assembly would decrease clotting times. No adverse effects were observed for up to 1:10 peptide/blood concentrations (Fig. 8c and Supplementary Table 5). In contrast, collagen showed a significant clot time reduction, and chelation of calcium prevented clotting altogether. Whole blood clotting kinetics were also measured in the absence of an activator to monitor the influence of progelator on hemostasis. Similarly, no statistical difference in clotting was observed between the vehicle and the highest progelator concentration (1:10) at any timepoint (Fig. 8d and Supplementary Fig. 21). In contrast, collagen and glass controls increased the clot rate.

RBC hemolysis in the presence of progelator was used to assess acute toxicity (Fig. 8e and Supplementary Table 6). Doses up to 1:10 revealed <5% hemolysis, which is below the limit for consideration as a hemolytic biomaterial^58^. Finally, a pro-thrombotic assay using platelet-poor plasma (PPP) was used to monitor non-platelet and phospholipid-specific effects on blood (Fig. 8f), such as potential to inhibit thrombin activity, prevent crosslinking of fibrinogen, or sequestration of calcium for decreased platelet activation. This assay has the advantage of monitoring the intrinsic pathway only, which is useful for studying surface contact activation in blood–biomaterial interactions. No changes to coagulation rate (slope), half-maximal coagulation time, and extent of coagulation (Supplementary Fig. 21) were observed in samples incubated with progelator up to doses of 1:100 peptide/blood.

With hemocompatibility verified, we applied the progelator in an initial proof-of-concept study in vivo. Enzyme-responsive progelator, KFDF_Cyclic,_ doped with a small amount of Rho-KFDF_Cyclic_ (5 mol%) for imaging, was injected into the infarct region 7 days post MI in a rat ischemia–reperfusion model (Fig. 8g). Assessment of hearts at 24 h post injection confirmed that progelator effectively gelled at the heart (Supplementary Figs. 22-23). Histological and fluorescence analysis of heart sections revealed hydrogel assembly occurred at the site of MI in all rats (Fig. 8h, i). No differences were observed in cardiomyocyte apoptosis or macrophage infiltration (Supplementary Figs. 24-25) between saline- and peptide-injected infarcts at 24 h post injection, suggesting that the activation and subsequent gelation of enzyme-responsive progelators did not increase the infarct inflammatory response.

## Discussion

The number of candidate biomaterials amenable to minimally invasive cardiac catheter delivery to the heart post MI is limited. Consequently, translation of promising scaffolds from preclinical to clinical trials has been rare. In this study, we examined a platform modification strategy to enable one promising class of hydrogels, SAPs, to be injectable via a cardiac injection catheter by conformationally constraining linear SAPs into macrocyclic progelators which resist self-assembly (Fig. 1). These progelators were further modified with a substrate recognition sequence for endogenously expressed MI-associated proteases. We took the approach of designing peptide sequences based on a known SAP, and then utilizing in silico modeling to provide preliminary insight into any modifications that may cause disruption of gelation (Fig. 2). These modified sequences were then prepared synthetically for testing. Subsequent experimental analysis of self-assembly characteristics and mechanical properties agreed well with these predictions, namely that sequence modifications did not interfere with self-assembly as β-sheets into fibrils and viscoelastic hydrogels (Fig. 3). It is hypothesized that any number of other SAP sequences used in biomedical applications could similarly be adapted to our structurally dynamic platform^59,60^.

The versatility of our platform is demonstrated through the functionalization of two different SAPs sequences, which exhibit disparate self-assembly mechanisms, and yet form progelators with identical responsiveness. Cyclic progelators synthesized in Fig. 4 lacked the capacity to gel when sterically constrained. However, they exhibited responsiveness to enzymes that are inherently active in the MI during both the acute inflammatory phase and fibrotic phase (Fig. 5). Despite the different cut sites recognized by MMP-9 catalytic domain, elastase, and thermolysin, similar secondary structure and nanoscale morphology changes were observed. Bulk cleavage of these sterically constrained materials resulted in healable viscoelastic hydrogels that were stable against excess proteolysis (Fig. 6).

The development of smart structurally dynamic and responsive materials for tissue engineering post MI is a relatively untapped field. With many scaffolds, the common issue remains that chemical modification (e.g., crosslinks, binding moieties, targeting motifs, or therapeutics drugs) can negatively impact material properties such as sample viscosity and gelation kinetics, which would preclude their use in catheters^61^. With our design, mechanical differences (e.g., stiffness or healing kinetics) in the resulting hydrogel would not affect the initial progelator formulation. Importantly, we provide a demonstration of low-viscosity peptide-based progelators for use in a cardiac injection catheter with little resistance to flow (Fig. 7). The results of these experiments demonstrate that the cyclic progelator acts as a non-Newtonian fluid for smooth catheter-based injection and still retains the capacity to solidify when acted upon by endogenously expressed inflammatory enzymes in the heart, suggesting this could provide a useful strategy for cardiac catheter delivery in vivo. While two types of materials have been delivered via catheter to the heart in clinical trials, namely alginate and a myocardial ECM hydrogel, our approach could have some unique advantages since it is a synthetic system that can be precisely tuned, is well defined, and has limited batch-to-batch variation.

With the potential for tissue leakage into the bloodstream, thrombogenicity and toxicity of transendocardially injected biomaterials is a concern. Synthetic modifications to the SAPs were shown to imbue no cytotoxicity to human CPCs and were hemocompatible and non-thrombogenic through a comprehensive assessment of whole blood clotting times, hemostatic kinetics, RBC hemolysis, and pro-thrombotic assays (Fig. 8). A dosing analysis shows no statistically significant difference in hemostasis due to increasing progelator concentration in whole blood. The extent of coagulation in PPP was only altered at the highest peptide dose which indicates that local high concentrations of peptide have an minor anti-coagulative effect on the intrinsic coagulation (contact-based) pathway; furthermore, the impact that our peptide does have on coagulation was shown to be independent of platelet-dependent thrombus formation, a key player in the pathogenesis of acute MI^62^. This conclusion is supported by the lack of change in pro-thrombotic profiles when incubated with collagen, which increases clotting rates only through the activation of platelet aggregation^63^. Given a ‘standard' clinical concentration of 1:10,000^56^, which assumes that no material was injected into the heart wall and instead escaped into the bloodstream, we observed no adverse effects on coagulation at this clinically relevant dose. These results indicate that this platform for generating an inert cyclic adduct of known SAPs should not result in unwanted blood interactions during delivery. Our platform enables facile delivery of a free-flowing progelator, and on-demand gelation at the site of inflammation (Fig. 8). In vivo analysis of KFDF_Cyclic_ progelator revealed successful gelation of activated hydrogel scaffolds in a rat MI model, without inducing an inflammatory response or cardiomyocyte apoptosis. As such, we have demonstrated a simplistic strategy that uses the naturally complex MI microenvironment for delivery and smart assembly in tissue. This proof-of-concept test paves the way for exploring the amenability of various other SAP systems (e.g., RAD16-II) with our progelator system for cardiac injection in vivo. Additionally, there exists the potential to use this platform in conjunction with various therapeutics. Herein, we observed that covalent conjugation of a rhodamine dye to cyclic progelators did not disrupt gelation. We envision a modular system to deliver and sustain any number of covalently bound therapeutics (e.g., small molecule drugs or short peptides). For larger therapeutics (e.g., cell therapies or growth factors), injecting a mixture with the cyclic progelators and subsequent encapsulation within locally activated networks might provide a means for targeted delivery without the need for complex synthetic chemistries. Ultimately, our simple strategy for structural control of self-assembling peptides could be employed for minimally invasive delivery of promising therapeutic peptides to other forms of injury or disease where MMPs are upregulated, including osteoarthritis^64^, cartilage tissue repair^41^, nerve damage^65^, and acute brain injury ^66^.

## Methods

### Peptide synthesis

Peptides were synthesized in an AAPPTec Focus XC peptide synthesizer. Peptides with C-terminal amides were synthesized on rink amide MBHA resin and peptides with C-terminal carboxylic acids were synthesized on Wang-OH resin using double coupling conditions for the first amino acid. HBTU (*N*,*N*,*N*′,*N*′-tetramethyl-O-(1H-benzotriazol-1-yl) uronium hexafluorophosphate) was used as the general coupling agent. When included, 5(6)-carboxytetramethyl rhodamine was incorporated during the synthesis at the N terminus. General peptide cleavage and deprotection was performed in 95:2.5:2.5 (%v/v) trifluoroacetic acid (TFA), triisopropyl silane, and H_2_O, respectively, for 2 h. Cleaved peptides were precipitated in cold anhydrous ether (3×) to yield solid crude. Semi-protected peptides containing Cys(acm) were purified prior to cyclization. Fmoc was temporarily used for unlabeled progelators to improve peak separation during purification and to monitor cyclization by high-performance liquid chromatography (HPLC) and electrospray ionization (ESI; Fig. 4b–e). To synthesize cyclic progelators, iodine was used to simultaneously deprotect Cys(acm) and initiate disulfide bond formation under dilute conditions to favor intramolecular macrocyclization. To a solution of semi-protected peptides (500 µM) in a mixture of acetic acid/methanol/H_2_O (1:16:4) was slowly added 0.1 M methanolic iodide until the yellow color persisted (~4–5 eq). The reaction was vigorously stirred at room temperature for 2 h and reaction completion was confirmed by LCMS. After 2 h of reaction, Amberlite IRA-400 Resin (chloride form) was stirred in the solution for 1 h to quench excess iodine and absorb reacted iodide ions. Filtrated was placed on a rotary evaporator to remove acetic acid and methanol. The remaining solution was diluted with H_2_O and lyophilized to a white powder. By HPLC no dimerization was observed. For cyclization kinetics analysis (Fig. 4f, h), the N-terminal Fmoc was temporarily left on unlabeled peptides for improved peak separation during purification.

### Peptide purification and analysis

Analytical scale RP-HPLC analysis of peptides was performed on a Jupiter Proteo90A Phenomenex column (150 × 4.60 mm) using a Hitachi-Elite LaChrom L2130 pump with ultraviolet–visible (UV–Vis) detector (Hitachi-Elite LaChrom L-2420) monitoring at 214 nm, 256 nm, 290 nm, and 565 nm. Gradients performed over 30 min. LCMS was performed with a Waters AQUITY UPLC System using a C-18 column over a 4 or 10 min gradient. Peptides were purified with a Jupiter Proteo90A Phenomenex column (2050 × 25.0 mm) on an Armen Glider CPC preparatory phase HPLC over a 43 min gradient to yield 90–95% purity. For all RP-HPLC assays and purifications, gradient solvent systems utilized Buffer A (H_2_O with 0.1% TFA) and Buffer B (acetonitrile with 0.1% TFA). For all LCMS assays, gradient solvent systems used Buffer A (H_2_O) and Buffer B (acetonitrile with 0.1% formic acid). Crude peptide was prepared for purification in 5:25:70 acetic acid/Buffer A/Buffer B via initial dissolution in acetic acid with sonication, followed by addition of acetonitrile and then H_2_O. Unless otherwise stated, peptides were purified using a gradient of 25–45% Buffer B over 30 min and 50 min for analytical HPLC and preparatory phase HPLC, respectively. Following purification, product was analyzed by ESI to verify identity (see Supplementary Fig. 3). Due to the large number of *cis*/*trans* configurational isomers that our cyclic progelators could adopt, and amphiphilic self-assembling nature of our peptides, peak resolution via HPLC was low in many instances. We relied on various mass spectrometry techniques (ESI, MALDI, HRMS, and tandem-MS) to verify purity of isolated materials.

### UV absorbance spectra

UV absorbance spectra of peptide labeling were measured on a ThermoScientific Nanodrop 2000c.

### Photographs of samples and illustrations

Photographs of vial flips, samples, and experimental setups were taken with an Android Samsung S7. Adapted clipart from Servier Medical Art is licensed under a Creative Commons Attribution 3.0 Unported License.

### Progelator sterilization and formulation for in vivo analysis

HPLC purified peptides were dialyzed with 1 kDa molecular weight cutoff tubing into milliQ H_2_O, sterile filtered through a 0.2 µm PES filter, and lyophilized to a powder. Peptide was reconstituted prior to injection with sterile pH 9 H_2_O to form a clear solution (11.11 mM), then diluted further with sterile 10× DPBS to a final concentration of 10 mM peptide in 1× DPBS (pH 7.4). Solutions for in vivo analysis contained 5 mol% rhodamine-labeled progelator (0.5 mM labeled + 9.5 mM unlabeled) for fluorescence imaging purposes.

### Transmission electron microscopy

Formvar/Carbon-coated 400 mesh Cu grids (Ted Paella, Inc.) were glow discharged for 90 s and spotted with 4–5 µL peptide sample (100 µM) and set for 5 min. Grids were washed with distilled H_2_O (10 drops), stained with 1% w/w uranyl acetate (3 drops), and wicked dry with filter paper. TEM images were acquired on an FEI Tecnai G2 Sphera at 200 kV.

### Experimental CD

Peptides were dissolved at a final concentration of 125 µM (labeled peptides) or 500 µM (unlabeled peptides) in 10 mM Tris buffer or H_2_O (for TCEP reduction only) at pH 7.4 to reduce signal interference seen with DPBS at lower wavelengths. UV–Vis CD was measured on a Jasco J-810 Spectropolarimeter to evaluate the secondary structure of peptide samples. Measurements were taken using the following settings: wavelength range = 190–260 nm, scanning speed = 50 nm min^−1^, response time = 2 s, data pitch = 1 nm, band width = 1 nm, accumulations = 3, pathlength = 1 mm, and temperature = 37 °C. Spectra are presented as an average of all accumulations. Sample voltage was monitored to verify the signal intensity did not exceed 800 mV within the wavelength range. Spectra were converted from units of millidegrees (mdeg) to molar ellipticity [θ] using the following equation:

1 $$\left[ \theta \right] = \frac{{{\mathrm{mdeg}} \ast M}}{{C \ast L \ast 10}},$$

where mdeg is the measured absorbance of circularly polarized light, *C* is concentration in g L^−1^, *M* is the mean residual weight in g mol^−1^, and *L* is the cell pathlength in cm.

### Computational modeling with FibPredictor

FibPredictor was utilized as a commercially available software for generating native-like amyloid fibril structures^42^. This software was chosen for our study due to its unique ability to reliably model all classes of amyloid fibrils starting from sequence-only input. The algorithm combines β-sheet model building, β-sheet replication, and symmetry operations with side-chain prediction and statistical scoring functions for computational predictions on amyloid fibril structures of self-assembling peptides utilized in this study. From each random model generated, a SCRWL internal scoring function was used by SCWRL4 to predict the energetically lowest side-chain orientations. This scoring function was used to calculate the minimal total energy of the entire model and output coordinates for the structure as a PDB file (see Supplementary Data) with predicted side chains using the same residue numbering and chain identifiers as the input structure. To identify the most energetically favorable candidate structures in the ensemble, both Amb_3b (computationally more efficient)^67^ and GOAP (more accurate)^68^ statistical scoring functions were used to calculate total energies of the protein structure. Reported energies are unitless as they are empirically derived scores, and thus it is not advisable to interpret results as kcal mol^−1^. Normalized GOAP scores are useful for comparing fibrils of different sizes. Three generations of modeling were performed with literature-recommended parameters shown in Supplementary Table 1. Results of top model for each peptide sequence from the third-generation analysis are reported in Supplementary Table 2. Corresponding structures and raw PDB files are provided in the main text Fig. 2 and as Supplementary Data. Output PDB files, containing the atomic coordinates of the chromophores, from top models were used to generate theoretical CD spectra with DichroCalc^45^.

### Theoretical CD spectra with DichroCalc

DichroCalc was used to calculate CD spectra using predicted PDB from FibPredictor. This web interface predicts secondary structure type using a variety of matrix method parameters, which have been derived from ab initio calculations. These parameters include the peptide chromophore in the far-UV, charge-transfer between neighboring peptide groups in the deep-UV, and aromatic side-chain chromophores with transitions in the near-UV. Full analysis CD spectra were output in units of molar ellipticity [θ] vs wavelength.

### In vitro enzyme cleavage of cyclic peptide progelator

Unless otherwise stated, enzyme cleavage experiments were performed on progelator at a final concentration of 500 µM in 1× enzyme cleavage buffers. MMP-9 cleavages were performed at 1:1000 enzyme/substrate molar ratio in 1× buffer (50 mM Tris-HCl, 200 mM NaCl, 5 mM CaCl_2_, 1 mM ZnCl_2_, pH 7.5) for 5 h. Elastase cleavages were performed at 1:250 enzyme/substrate molar ratio in 1× buffer (100 mM Tris-HCl, 0.2 mM NaN_3_, pH 8.0) and thermolysin (50 mM Tris-HCl, 0.5 mM CaCl_2_) for 5 h. Thermolysin cleavages were performed at 1:4500 enzyme/substrate molar ratio in 1× buffer (1× DPBS, pH 7.4) for 15 min. Analysis of unlabeled cyclic peptide progelator cleavage by CD (Fig. 6f) was conducted with serials additions of thermolysin (4 × 1:18,000 enzyme/substrate) over a period of 73 h in 10 mM Tris buffer. Control samples utilized denatured enzymes under the same conditions. MMP and elastase were heat denatured with 10 min of incubation at 65 °C. Thermolysin was inactivated by incubation with 10% (v/v) EDTA (0.5 M, pH 8.0).

### Rheological characterization

All peptide samples were prepared at 10 mM peptide, except for those used to assess the effect of SAP functionalization with respect to weight (15 mg mL^−1^). Viscous and viscoelastic properties were assessed using a stress-controlled rheometer (TA Instruments AR-G2) equipped with a Peltier plate to control temperature and a 20 mm diameter parallel plate geometry. Unless otherwise stated all measurements were taken at an angular frequency of 2.5 rad s^−1^, strain of 0.5%, and temperature of 37 °C. Measurements were performed with a gap height of 1000 µm and repeated three times (except for step-strain and time course measurements) to ensure reproducibility. To prevent water evaporation, mineral oil was wrapped around the edge of the geometry at the air–sample interface. For viscoelastic measurements the apparatus was used in oscillatory mode. To ensure the measurements were made in the linear viscoelastic regime, strain sweeps were performed between 0.05 and 50% strain and showed no variation in storage (G′) and loss (G″) moduli up to a strain of 0.5%. The dynamic moduli of the hydrogels were measured as a function of frequency in the range 0.25–100 rad s^−1^ at a strain of 0.5%. Continuous step-strain oscillations were used to monitor hydrogel healing through disruption (3 min, 100% strain) and recovery (15 min, 0.5% strain) cycles (*n* = 3). For viscosity measurements the apparatus was used in steady-state flow mode. The viscosity of the samples was measured as a function of shear rate in the range 0.1–10 s^−1^ (5% tolerance). For time course measurements of enzyme activation, a stock of cyclic peptide (1 mL, 10 mM, 1× DPBS, pH 7.4) was prepared and incubated at 37 °C. A sample from this stock was applied to the rheometer for storage and loss moduli measurements (2.5 rad s^−1^, 0.5% strain) over a 35–40 min period. This sample was removed and replaced with an identical sample mixed via pipetting for 5 s with thermolysin (1:4500 enz/peptide) followed by immediate addition to the rheometer. Delay time between enzyme addition and rheology measurements was ~30 s.

### In vitro catheter injections and effect on assembly

Peptide solutions (0.6–0.8 mL) were prepared at 10 mM in 1× DPBS (pH 7.4) and loaded into a 1 mL Leur Lock syringe attached to a syringe pump set to a flow rate of 0.6 mL min^−1^. Peptide was injected through the 27 G inner nitinol tubing of a MyoStar catheter that was immersed in a 37 °C water bath.

### Hemocompatibility analysis

For all measurements, peptide stocks in 1× DPBS were prepared such that the final concentrations (v/v) in blood or plasma were 1:10, 1:100, 1:500,1:1000, 1:5000, 1:10,000, and 1:20,000 (peptide/fluid volume). Controls with collagen as a procoagulant initial platelet-adhesive surface (0.095 mg mL^−1^ or 1:240 dilution) or with calcium chelated by sodium citrate utilized 1× DPBS as a vehicle to ensure consistent blood dilution in all experiments. Peptide stocks, whole human blood, isolated RBCs, and PPP were warmed to 37 °C immediately prior to use. Plate reader measurements were conducted on an EnSpire Multimode Plate Reader with 96-well tissue culture plates (TCP). All blood studies were done in compliance with the Northwestern University Bloodborne Pathogens Program. Unspun human whole blood, Na citrate, (BSC, LS 2402453A), was processed under US FDA registration #2577632 and pre-screened for viral contaminants (Title 21-CFR PART 610.40).

### ACT with whole human blood

A Hemochron 801 instrument calibrated with an electronic system verification (ESV) device was used to measure ACT of whole human blood. ACTs were determined using recalcified citrated whole human blood to minimize variability in starting time points for clotting in all assays. To each Hemochron P214 tube with glass beads was added 4 µL CaCl_2_ (1.1 M) and 36 µL peptide stock or additive (12.2× final blood concentration). Samples were mixed thoroughly for 30 s to soak the glass beads and incubated for 30 s at 37 °C. Citrated whole human blood (400 µL) was then added (*t* = 0 s), mixed by hand for 10 s, and added to the instrument. Time points at which the magnet was displaced by clot formation were recorded by the instrument. Collagen (0.095 mg mL^−1^) was used as a positive control to decrease clotting time. Vehicle (1× DPBS) served as a standard for blood without additive. Samples without calcium, serving as the negative control, exceeded instrument maximum time range (>1500 s). Each experiment was performed *n* = 6 times with averages and standard error of the mean (SEM) plotted.

### Whole blood hemostasis kinetics

Changes to time-dependent hemostasis were monitored using a non-activated whole blood clotting assay. Briefly, 100 µL of citrated whole human was mixed with 10 µL of peptide or collagen stock (11.5× final blood concentration) and 5 µL of CaCl_2_ (230 mM). Collagen (0.095 mg mL^−1^) and glass coverslips were used as positive controls to increase clotting rates from an additive and contact initiated perspective, respectively. Samples without calcium, where no clot was observed at any timepoint, served as negative controls. Aliquots (100 µL) were transferred to a 12-well TCP, covered, and incubated at room temperature for 5, 15, 30, and 45 min. Each sample was prepared in triplicate. At the end of each timepoint, RBCs not caught in the thrombus were lysed by gently adding 3 mL distilled H_2_O and incubating for 5 min. Each well was sampled (200 µL), taking care not to disturb the clot, and transferred to a 96-well plate for analysis. Released hemoglobin from lysis was detected by measuring the absorbance at 405 nm (height 4.0 mm, 100 flashes) using a plate reader. Extent of clotting is inversely proportional to measured absorbance. Averages (*n* = 3) and SEM are plotted.

### Hemolysis assay

RBCs were isolated by centrifugation of 40 mL citrated whole human blood at 500 × *g* for 5 min, followed by gentle washes with 150 mM NaCl (×1) and 1× DPBS (×3). Isolated RBCs were diluted 1:50 into 1× DPBS (pH 7.4) and lightly agitated to prevent settling. Briefly, 190 µL of dilute RBCs and 10 µL of peptide stock or additive (20× final concentration) were mixed and added to a 96-well plate (*n* = 4 repeats), covered, and incubated at 37 °C for 1 h. Plates were centrifuged at 500 × *g* to pellet intact RBCs using a centrifuge equipped with a microplate rotor. Supernatant (100 µL) was transferred to a new 96-well plate, taking care not to disturb the pellet. Absorbance of the supernatant was measured at 540 nm to detect released hemoglobin. To calculate % hemolysis, absorbances were corrected for background absorbance from untreated vehicle and then normalized to 1% Triton X-100-treated RBCs to represent 100% hemolysis. %Hemolysis was calculated according to the following equation:

2 $${\mathrm{\% Hemolysis}} = \frac{{\left( {{\mathrm{Abs}}_{{\mathrm{experiemental}}}} \right) - \left( {{\mathrm{Abs}}_{{\mathrm{negative}}\,{\mathrm{control}}}} \right)}}{{\left( {{\mathrm{Abs}}_{{\mathrm{positive}}\,{\mathrm{control}}}} \right) - \left( {{\mathrm{Abs}}_{{\mathrm{negative}}\,{\mathrm{control}}}} \right)}} \times 100,$$

where Abs_experiemental_ is the average well absorbance pertaining to RBCs pertaining to the additive sample being analyzed, Abs_negative control_ is the average well absorbance for RBCs incubated with vehicle (1× DPBS), and Abs_positive control_ is the average well absorbance for RBCs containing 1% Triton X-100.

### Pro-thrombotic assays

PPP was isolated from citrated whole human blood through two rounds of centrifugation at 2000 × *g* for 10 min and collection of the upper two-third layer. Briefly, 100 µL of PPP, 50 µL of peptide stock or additive (4× final concentration), and 50 µL of CaCl_2_ (50 mM) were mixed and added to a 96-well plate and incubated at 37 °C for kinetics measurements. For each sample, *n* = 6 repeats were conducted. Coagulation profiles were obtained by measuring well absorbance at 405 nm at 30 s intervals for 60 min. Onset to clotting was detected as a sharp increase in sample turbidity.

### In vivo studies

All animal experiments were conducted in accordance with the guidelines established by the Institutional Animal Care and Use Committee at the University of California, San Diego (UCSD), and the Association for the Assessment and Accreditation of Laboratory Animal Care and approved by the Institutional Animal Care and Use Committee at the UCSD (A3033-01). Female Sprague-Dawley rats (225–250 g) were used in all studies.

### Intramyocardial injections of peptide into infarcted rats

MI was performed via 35 min ischemia–reperfusion and intramyocardial injections were performed under isoflurane^69^. Briefly, cyclic peptide (75 µL, 0.75 µmol, 10 mM) was administered as a single injection into the infarct with a 27 G needle at 7 days post MI. Animals were euthanized with an overdose of pentobarbital (200 mg kg^−1^) at 24 h post injection (*n* = 5).

### Cryosectioning, histology, and immunofluorescence imaging

After euthanasia, hearts were resected, fresh frozen in TissueTek OCT, and cryosectioned for histological analysis. Slides were either stained with hematoxylin and eosin (H&E) to identify the infarct region or stained for immunofluorescence analysis. H&E slides were imaged on an Aperio ScanScope CS^2^ at ×20 magnification. For immunofluorescence, tissue sections were permeabilized in acetone (−20 °C) for 1.5 min and blocked with 2% bovine serum albumin and 0.3% Triton X-100 in 1× PBS. Myocardium was labeled using mouse anti-rat α-actinin antibody (1:800 dilution in blocking buffer, 1 h of incubation, Sigma A7811) and an Alexa Fluor 488 goat-anti-mouse IgG secondary antibody (1:500 dilution in blocking buffer, 30 min of incubation, Thermo Fisher A-11001). Nuclei were labeled using Hoechst 33342 (1:10,000 dilution in deionized water, 10 min of incubation, Life Technologies). Tissue sections were mounted with Fluoromount (Sigma) and imaged using a Leica Ariol slide scanner with Ariol software at ×20 magnification.

### Human cardiac progenitor cell culture

Human fetal CPCs were isolated from tissue harvested under standard informed consent procedures and prior approval of the ethics committee of the University Medical Center Utrecht. All cell studies were done in compliance with the UC San Diego Human Research Protections Program. CPCs were isolated from human fetal hearts for selection with Sca-1^+^ magnetic bead sorting (Miltenyi Biotech, 130-091-176) and cultured on 0.1% gelatin in growth media. Growth media consists of 25% EGM-2 (Cambrex, CC-3156) supplemented with EGM-2 Single Quots (Cambrex, CC-4176), 10% fetal bovine serum, 1× penicillin/streptomycin (Sigma, P4458), and 1× minimum essential medium (MEM) non-essential amino acids (BioWhittaker, BE13-114E) in M199 (BioWhittaker, BE12-119F). Cells at passages 3–5 after isolation were tested for mycoplasma, showing no contamination. Cells at passages 17–21 were used for all experiments.

### Cytotoxicity assays

Material acute cytotoxicity was tested using human CPCs. The cells were stained with calcein-AM cell viability dye (25 nm; eBioscience) for 30 min and 500,000 cells were resuspended in 50 µL of each peptide (10 mM). After 15 min, 1 mL of growth media was added to the peptide-cell mixture and 50 µL was transferred into a 24-well plate for imaging. Five to six images were taken per condition using a fluorescence microscope (Carl Zeiss, Dublin, CA, USA). Cells resuspended in media only were used as a control. The percentage of viable cells was determined by counting the stained cells over the total number of cells. Three experiments were performed (*n* = 8–12 repeats in duplicate). Alternatively, CPCs were also cultured up to 4 days with or without peptides, and cytotoxicity was evaluated by an Alamar blue metabolic activity assay. In all, 3500 hCPCs were plated into each well of a 96-well plate in growth media. At 24 h after cell seeding, baseline metabolic activity was taken by incubating the cells in Alamar blue (1:10 in medium, Invitrogen) for 3 h and analyzing at 550/585 nm with a Synergy™ 4 Multi-Mode Microplate Reader (Biotek). Peptide was added to cells and cultured for 4 days. Alamar blue assay was repeated after 24 and 96 h, and growth media was used as control (*n* = 3 repeats in quadruplicate). Metabolic activity reported as fold increase vs baseline values.

### Statistical analysis

All statistical results are expressed as mean ± SEM. Ordinary one-way analysis of variance (ANOVA) tests were used for multiple comparisons of the mean in each group with that of the standard. Tukey's corrections with 95% confidence intervals and significance were used. Two-way ANOVA tests without matching were used for multiple comparisons of the mean in each group at each timepoint with that of the standard. Holm–Sidak test was used for multiple comparisons. Statistical significance was defined as follows: ns (*p* > 0.05), *p ≤ 0.05, **p ≤ 0.01, ***p ≤ 0.001, and ****p ≤ 0.0001.

### Reporting summary

Further information on experimental design is available in the Nature Research Reporting Summary linked to this article.

## Supplementary information

Supplementary Information

Description of Additional Supplementary Files

Supplementary Movie 1

Supplementary Data 1

Supplementary Data 2

Supplementary Data 3

Supplementary Data 4

Supplementary Data 5

Supplementary Data 6

Reporting Summary

## Data Availability

The authors declare that all data supporting the findings of this study are available within the article and its Supplementary Information files or from the corresponding authors on reasonable request.
