# Supplementary material for: Enzyme-responsive progelator cyclic peptides for minimally invasive delivery to the heart post-myocardial infarction
Source: Nat Commun. 2019 Apr 15;10:1735. doi: 10.1038/s41467-019-09587-y (PMC6465301; doi:10.1038/s41467-019-09587-y)
Supplement: Supplementary file 1 — Supplementary Information [file 41467_2019_9587_MOESM1_ESM.pdf]

Supplementary Information

Enzyme-responsive progelator cyclic peptides for minimally invasive delivery to the heart post-myocardial infarction

A. S. Carlini, et al.

Supplementary Methods

General information. Amino acids used in Fmoc SPPS were purchased from AAPPTec and NovaBiochem. All other synthetic materials were obtained from Sigma-Aldrich and used without further purification unless otherwise noted. MMP-9 (recombinant human catalytic domain) (BML-SEro360) was acquired from Enzo Life Sciences, as a 1.0 mg mL^{-1} solution at 40 U μL^{-1} in 50mM TRIS, pH 7.5, 1mM $CaCl_2$, 300mM NaCl, 5μM $ZnCl_2$, 0.1% Brij-35, and 15% glycerol. Porcine pancreatic elastase (3246821000U) was acquired from EMD Millipore, as a lyophilized powder (22 U mg^{-1}). Thermolysin (V4001) was acquired from Promega, as a lyophilized powder. Unspun whole human blood (citrated, non-heparinized) was obtained from Biological Specialty Corporation (LS23 95099) and stored at 1-6 °C.

Mass spectrometric (MS) analysis of peptides. General mass spectra were obtained at the UCSD Chemistry and Biochemistry Molecular Mass Spectrometry Facility using a Micromass Quattro Ultima Triple Quadrupole Electrospray Ionization (ESI) mass spectrometer or Bruker Biflex IV MALDI-TOFMS using HCCA (1:1 v/v) as the matrix. HRMS on a Thermo LTQ Orbitrap XL MS and Tandem-MS on a Thermo LCQdeca-MS were collected for cyclic progelators to discern conformation as cyclic or linear. DisConnect software[1] was used to unambiguously characterize disulfide connectivity. The following settings were used: MS^2 fragmentation ions, monoisotopic mass calculations, tolerance ± 3Da, normal peptide fragments (no losses), and calculations based on ion-trap filtering.

Static and dynamic light scattering (SLS and DLS). Light scattering measurements were performed on a Wyatt DynaPro NanoStar at 37 °C (λ = 657 nm). For each DLS measurement, 10 acquisitions for 10 sec were taken. Samples were prepared at 1 mg mL^{-1} in either H_2O, 1x DPBS, or 295 mM sucrose (pH 7.4). Experiments with enzyme activation used 1:4500 enzyme/substrate molar ratio.

Thermolysin cleavage for MALDI-MS. Progelators dissolved at 500 μM peptide in H_2O were treated with thermolysin (1:4500 enzyme/substrate) for 300 min at 37 ºC.

CD68+ tissue staining for macrophages. Tissue sections were fixed with 4% paraformaldehyde (PFA) for 5 min and blocked with 0.3% Triton X-100 in 1x PBS to permeabilize. Macrophages were labeled with mouse-anti-rat CD68+ primary antibody (1:100 dilution in staining buffer, Biorad MCA341R) for 1 hr at room temperature and incubated 24 hr at 4 ºC. Alexa Fluor 488 goat-anti-mouse secondary antibody (1:600 dilution in staining buffer, Thermo Fisher, A-11001) was incubated with agitation for 1 hr at room temperature and 12 hr at 4 ºC. Nuclei were labeled using Hoechst 33342 (1:10,000 dilution in DI water, Life Technologies) for 10 min at room temperature. Tissue sections were mounted with Fluoromount (Sigma) for imaging. Staining buffer was 10% donkey serum, 0.1% Triton X-100, and 3% BSA in 1x PBS.

Cleaved caspase-3 tissue staining for cardiomyocyte apoptosis. Tissue sections were fixed with acetone for 1.5 min and blocked with staining buffer for 20 min. Myocardium and cleaved caspase-3 were labeled using mouse-anti-rat α-actinin (1:800 dilution in staining buffer, Sigma A7811) and rat-anti-rabbit (1:200 dilution in staining buffer, Cell Signaling 9664) primary antibodies, respectively, overnight at 4 ºC for 20 hr. Alexa Fluor 488 goat-anti-mouse (1:600 dilution in staining buffer, Thermo Fisher) and Alexa Fluor 647 goat-anti-rabbit secondary antibody (1:500 dilution in staining buffer, Thermo Fisher, A-31573) secondary antibodies were incubated for 1.5 hr at room temperature. Nuclei were labeled using Hoechst 33342 (1:10,000 dilution in DI water, Life Technologies) for 10 min at room temperature. Tissue sections were mounted with Fluoromount (Sigma) for imaging. Staining buffer was 5% goat serum, 0.3% Triton X-100, and 1% BSA in 1x PBS.

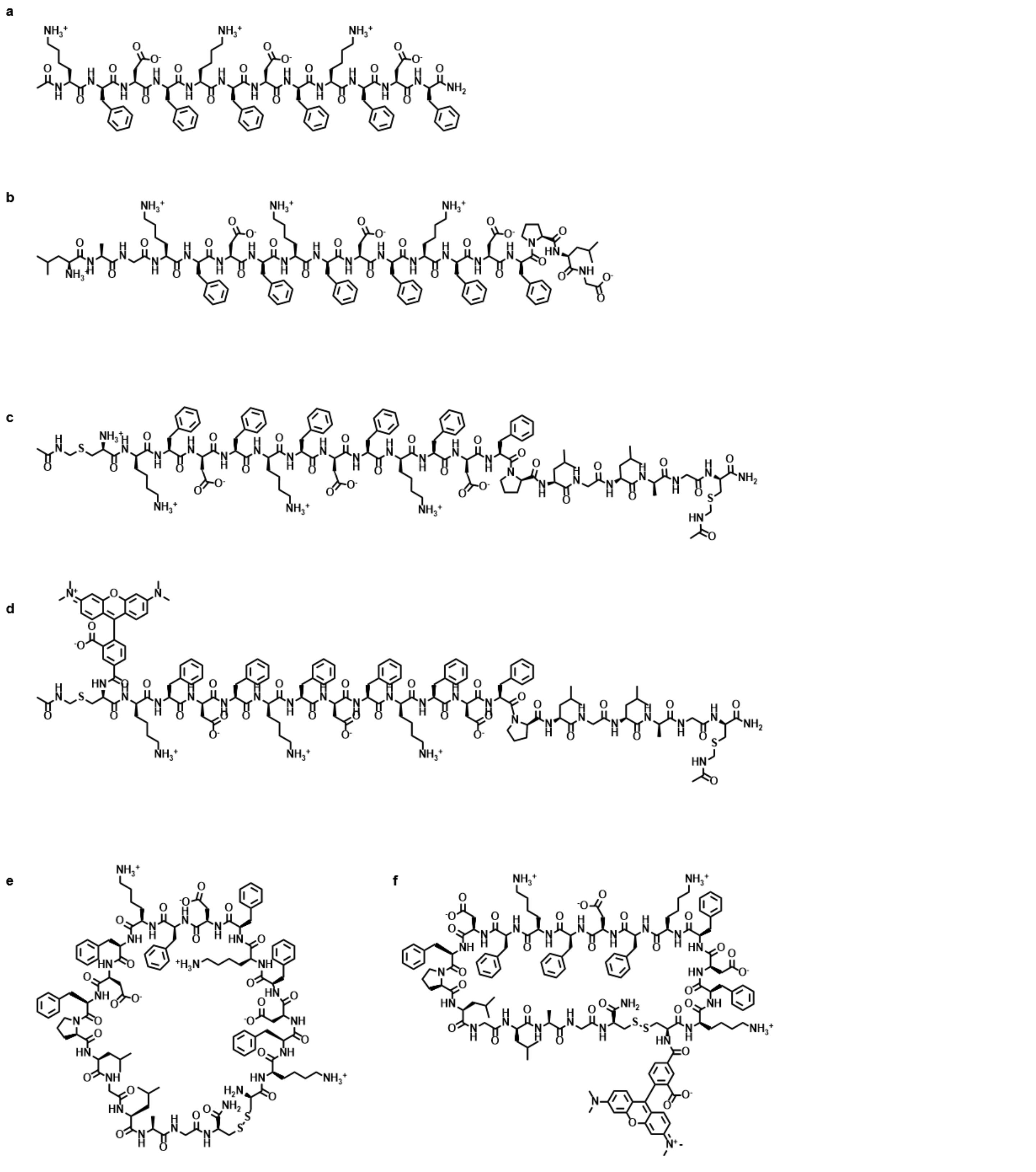

Supplementary Fig. 1. Chemical structures of KLDL peptides synthesized. a-f, KLDL (a), KLDL$_{\text{Control}}$ (b), KLDL$_{\text{Linear}}$ (c), Rho-KLDL$_{\text{Linear}}$ (d), KLDL$_{\text{Cyclic}}$ (e), and Rho-KLDL$_{\text{Cyclic}}$ (f).

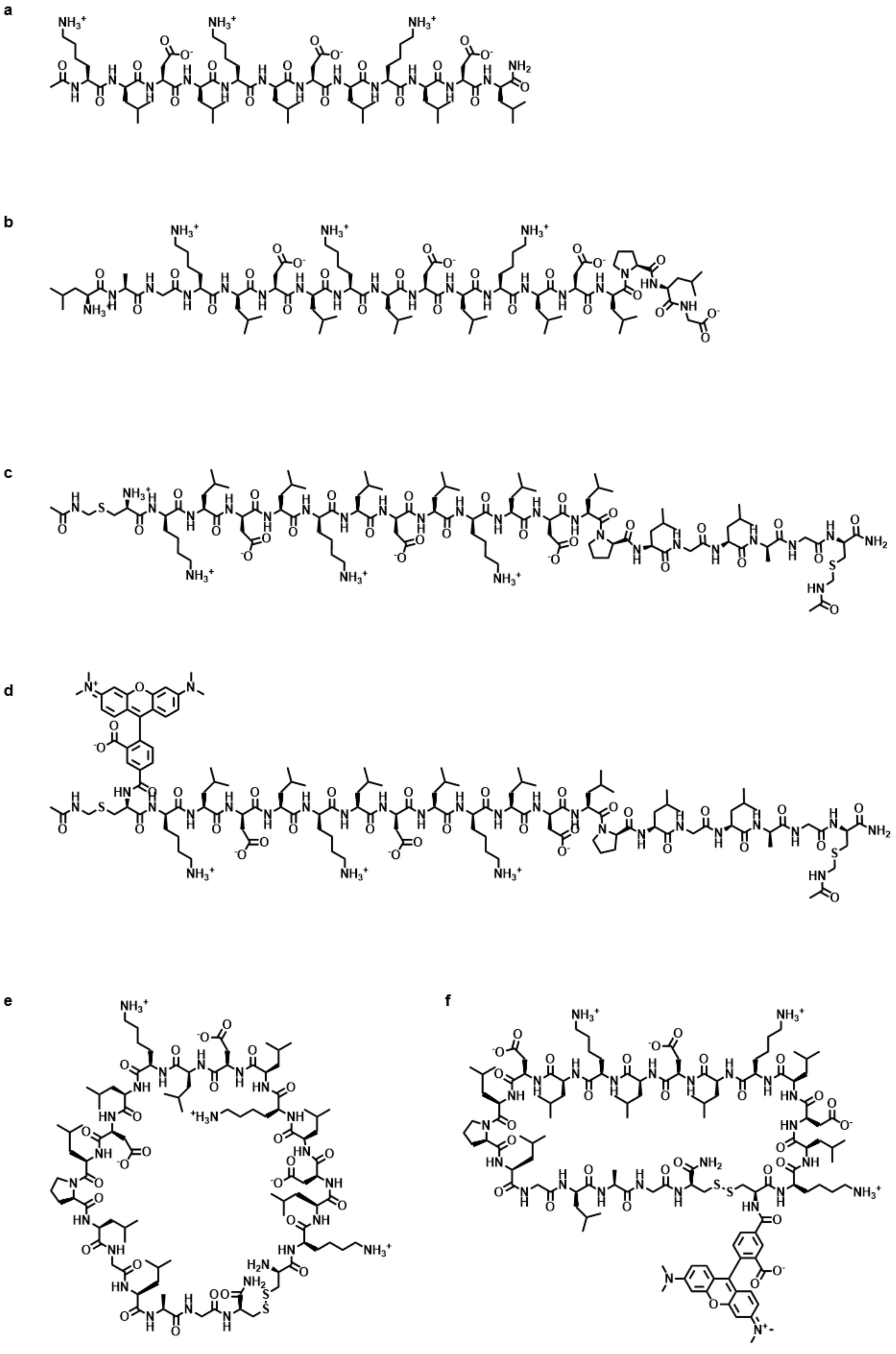

Supplementary Fig. 2. Chemical structures of KFDF peptides synthesized. a-f, KFDF (**a**), KFDF$_{\text{Control}}$ (**b**), KFDF$_{\text{Linear}}$ (**c**), Rho-KFDF$_{\text{Linear}}$ (**d**), KFDF$_{\text{Cyclic}}$ (**e**), and Rho-KFDF$_{\text{Cyclic}}$ (**f**).

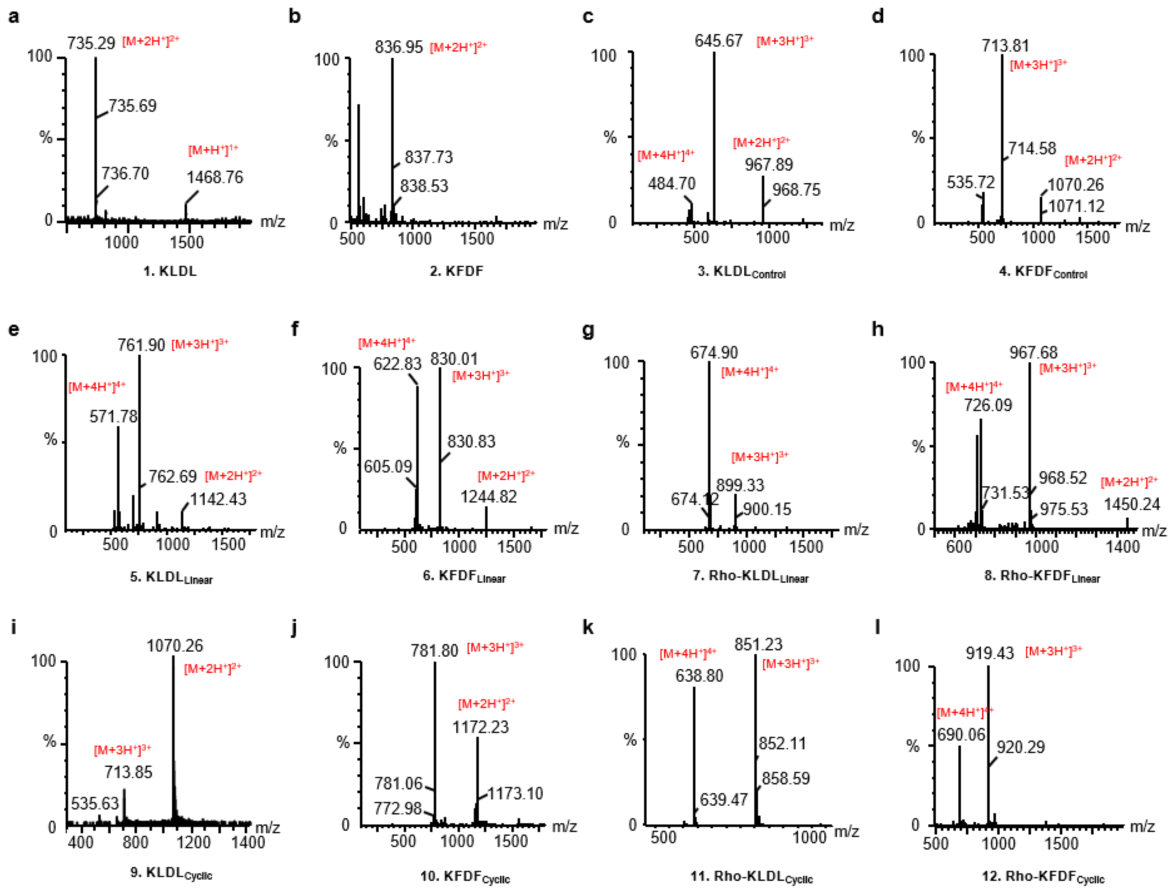

m

	Peptide Name	Peptide Sequence	Expected MW (pH < 3)	Observed m/z
1	KLDL	Ac-KLDLKLDLKLDL-conh$_2$	1470.84 g/mol	735.29 m/z $[M+2H^+]^{2+}$
2	KFDF	Ac-KFDFKFDFKFDF-conh$_2$	1674.94 g/mol	836.95 m/z $[M+2H^+]^{2+}$
3	KLDL$_{Control}$	H$_2$n-LAGKLDLKLDLKLDLPLG-cooh	1938.4064 g/mol	645.67 m/z $[M+3H^+]^{3+}$
4	KFDF$_{Control}$	h$_2$n-LAGKFDFKFDFKFDFPLG-cooh	2143.52 g/mol	713.81 m/z $[M+3H^+]^{3+}$
5	KLDL$_{Linear}$	h$_2$n -C(acm)LAGKLDLKLDLKLDLPLGC(acm)-conh$_2$	2286.87 g/mol	761.90 m/z $[M+3H^+]^{3+}$
6	KFDF$_{Linear}$	h$_2$n -C(acm)LAGKFDFKFDFKFDFPLGC(acm)-conh$_2$	2490.97 g/mol	830.01 m/z $[M+3H^+]^{3+}$
7	Rho-KLDL$_{Linear}$	Rho-C(acm)LAGKLDLKLDLKLDLPLGC(acm)-conh$_2$	2698.30 g/mol	674.90 m/z $[M+4H^+]^{4+}$
8	Rho-KFDF$_{Linear}$	Rho-C(acm)LAGKFDFKFDFKFDFPLGC(acm)-conh$_2$	2902.41 g/mol	967.68 m/z $[M+3H^+]^{3+}$
9	KLDL$_{Cyclic}$	h$_2$n-*CKLDLKLDLKLDLPLGLAGC*-conh$_2$	2141.68 g/mol	713.85 m/z $[M+3H^+]^{3+}$
10	KFDF$_{Cyclic}$	*CKFDFKFDFKFDFPLGLAGC*-conh$_2$	2345.79 g/mol	781.80 m/z $[M+3H^+]^{3+}$
11	Rho-KLDL$_{Cyclic}$	Rho-*CKLDLKLDLKLDLDFPLGLAGC*-conh$_2$	2555.14 g/mol	851.23 m/z $[M+3H^+]^{3+}$
12	Rho-KFDF$_{Cyclic}$	Rho-*CKFDFKFDFKFDFPLGLAGC*-conh$_2$	2759.24 g/mol	919.43 m/z $[M+3H^+]^{3+}$

Supplementary Fig. 3. ESI mass spectra and sequences of peptides synthesized. a-l, Spectra with accompanied m/z species identifications (shown in red) for unmodified SAPs, functionalized SAPs, and progelators in study. (a) KLDL, (b) KFDF, (c) KLDL$_{Control}$, (d) KFDF$_{Control}$, (e) KLDL$_{Linear}$, (f) KFDF$_{Linear}$, (g) Rho-KLDL$_{Linear}$, (h) Rho-KFDF$_{Linear}$, (i) KLDL$_{Cyclic}$, (j) KFDF$_{Cyclic}$, (k) Rho-KLDL$_{Cyclic}$, and (l) Rho-KFDF$_{Cyclic}$. m, Corresponding sequences with expected molecular weight (MW) and observed m/z values. * indicates a disulfide bond between neighboring cysteines.

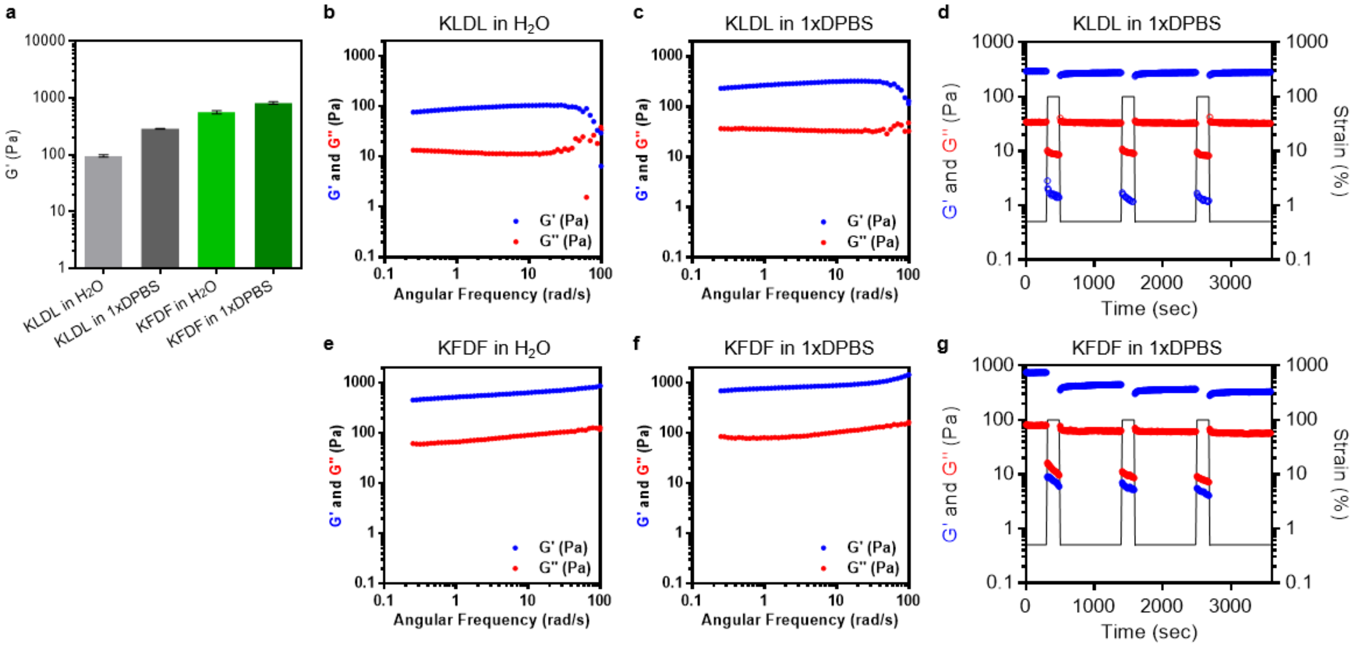

Supplementary Fig. 4. Rheological properties of KLDL and KFDF. Unmodified linear SAP compared with respect to molar concentration (10 mM) present as porous networks hydrogel networks with rehealable properties. Stiffer gels obtained when Phe substituted for Leu in the KFDF SAP. **a**, Storage moduli (G') for KLDL and KFDF SAPs in H_2O or 1xDPBS show increasing stiffness with increasing saline content and peptide hydrophobicity. Angular frequency 2.5 rad s^{-1}, 0.5% strain. **b-c**, Frequency sweeps (100-0.25 rad s^{-1}, 0.5% strain) of KLDL in H_2O and 1xDPBS. **d**, Step-strain oscillation analysis of KLDL in 1xDPBS. **e-f**, Frequency sweeps (100-0.25 rad s^{-1}, 0.5% strain) of KFDF in H_2O and 1xDPBS. **g**, Step-strain oscillation analysis of KFDF in 1xDPBS. KLDL and KFDF SAPs show rapid and repeat healing capacity of hydrogels (G'>G'') following complete network disruption (G''>G') at high strains. Viscoelastic storage (G') and loss (G'') moduli are plotted as a function of time with step cycles at 100 % destructive strain (t=3 min) followed by 0.5% recovery strain (t=15 min) to monitor healing. Hydrogels prepared at 10 mM in 1x DPBS. Angular frequency 2.5 rad s^{-1}.

.

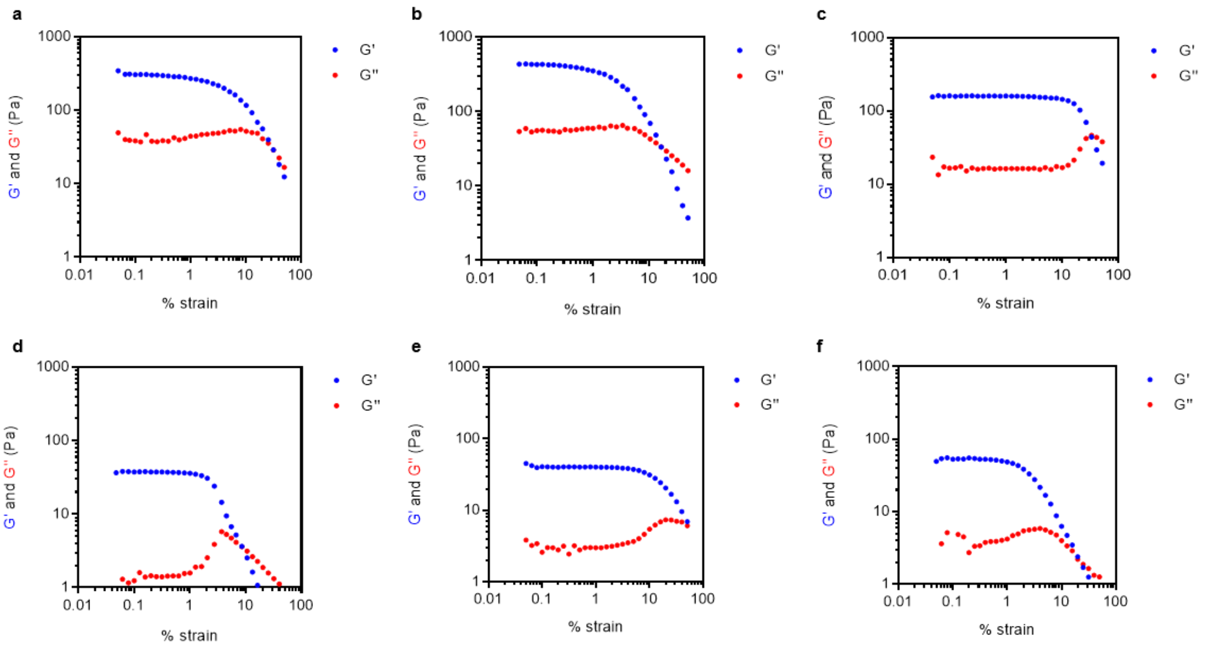

Supplementary Fig. 5. Strain sweeps of SAPs. a-e, Storage (G') and loss (G'') moduli were measured as a function of strain (0.5 – 50%) to identify the linear viscoelastic region (LVR) and appropriate rheological measurement conditions for all SAPs. Corresponding plots of (**a**) KLDL, (**b**) KFDF, (**c**) $KFDF_{Control}$, (**d**) $KFDF_{Linear}$, (**e**) $KFDF_{Cyclic}$ + thermolysin, and (**f**) $KLDL_{Cyclic}$ + thermolysin. A strain of 0.5% lies within the LVR for these strain sweeps and was chosen for all rheological measurements in this study. SAPs were prepared at 15 mg mL^{-1} in 1x DPBS. Angular frequency 2.5 rad s^{-1}.

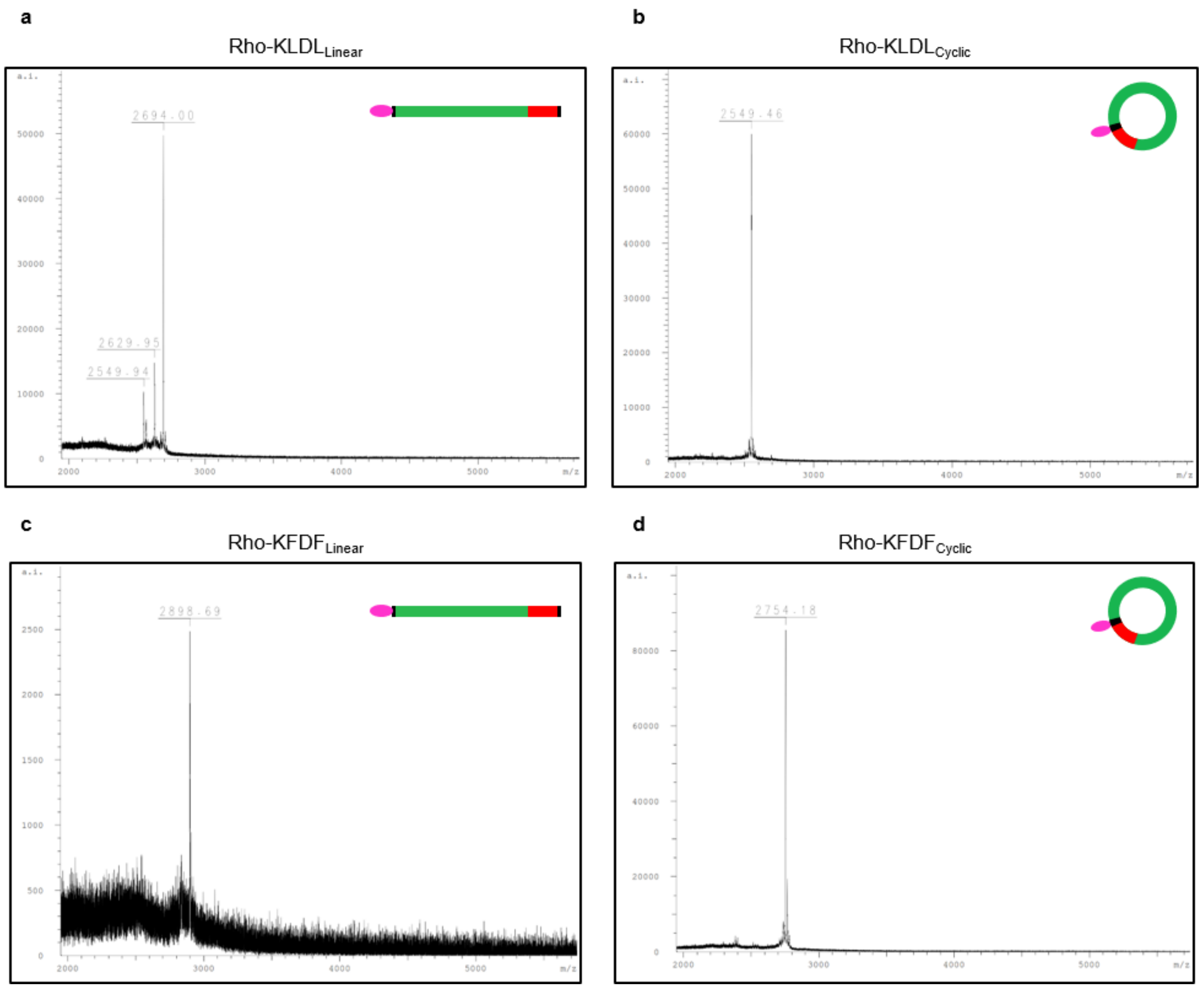

Supplementary Fig. 6. MALDI of labeled progelators before and after cyclization. a-b, Rho-KLDL$_{Linear}$ (**a**) and resulting cyclization product (**b**)Rho-KLDL$_{Cyclic}$. Masses are 2694.00 m/z [M+H$^+$]$^{1+}$ and 2549.46 m/z [M+H$^+$]$^{1+}$, respectively. **c-d**, Rho-KFDF$_{Linear}$ (**c**) and resulting cyclization product (**d**)Rho-KFDF$_{Cyclic}$. Masses are 2989.69 m/z [M+H$^+$]$^{1+}$ and 2754.18 m/z [M+H$^+$]$^{1+}$, respectively.

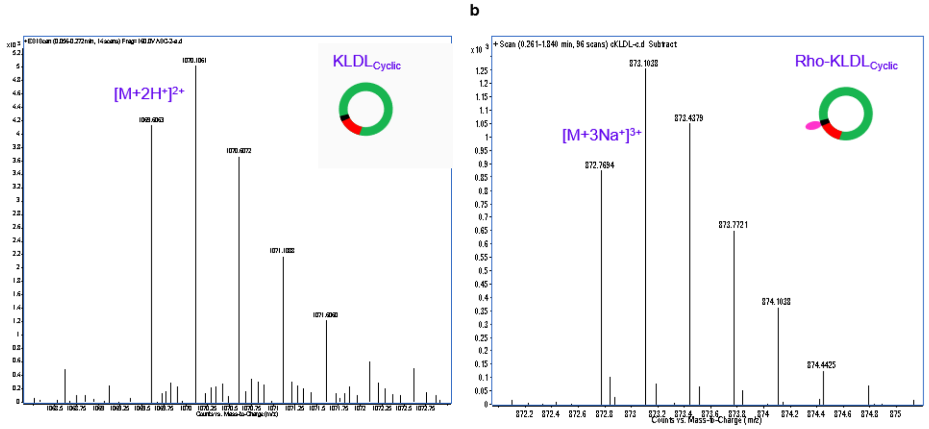

c

	Conformation	Theo. Mass (m/z)	Mass Measured (m/z)	Delta (ppm)	Composition
Unlabeled	open	1070.6153			$[C_{96}H_{172}N_{24}O_{26}S_2]^{2+}$
	closed	1069.6074	1069.6063	-1.0	$[C_{96}H_{170}N_{24}O_{26}S_2]^{2+}$

	Conformation	Theo. Mass (m/z)	Mass Measured (m/z)	Delta (ppm)	Composition
Labeled	open	873.4420			$[C_{121}H_{190}N_{26}O_{30}S_2Na_3]^{3+}$
	closed	872.7701	872.7694	-0.8	$[C_{121}H_{188}N_{26}O_{30}S_2Na_3]^{3+}$

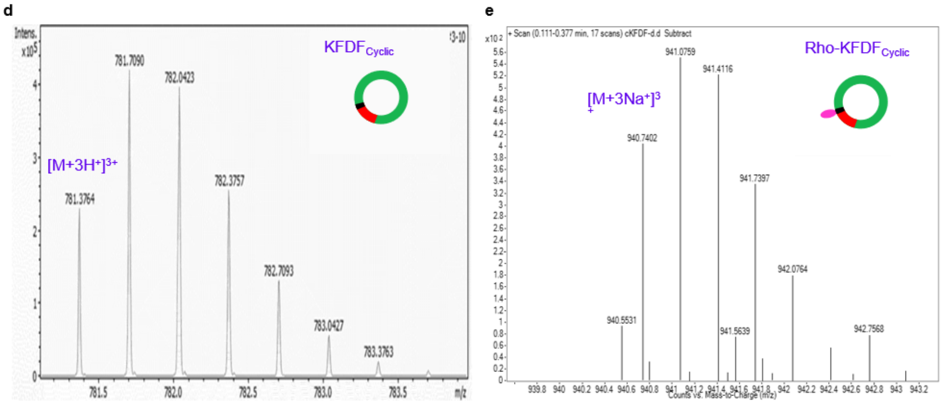

f

	Conformation	Theo. Mass (m/z)	Mass Measured (m/z)	Delta (ppm)	Composition
Unlabeled	open	782.0480			$[C_{114}H_{161}N_{24}O_{26}S_2]^{3+}$
	closed	781.3761	781.3764	0.4	$[C_{114}H_{159}N_{24}O_{26}S_2]^{3+}$

	Conformation	Theo. Mass (m/z)	Mass Measured (m/z)	Delta (ppm)	Composition
Labeled	open	941.4107			$[C_{139}H_{178}N_{26}O_{30}S_2Na_3]^{3+}$
	closed	940.7387	940.7402	1.5	$[C_{139}H_{176}N_{26}O_{30}S_2Na_3]^{3+}$

Supplementary Fig. 7. HRMS unlabeled and labeled progelators. Analysis conducted to identify whether final progelator peptides were in closed cyclic or open linear conformations. **a-b**, HRMS spectra for (**a**) KLDL$_{Cyclic}$ and (**b**) Rho-KLDL$_{Cyclic}$. **c**, Corresponding summary of theoretical m/z, measured m/z, measurement error, and elemental compositions for opened and closed conformations. **d-e**, HRMS spectra for (**d**) KFDF$_{Cyclic}$ and (**e**) Rho-KFDF$_{Cyclic}$. **f**, Corresponding summary of theoretical m/z, measured m/z, measurement error, and elemental compositions for opened and closed conformations. All measurements reveal closed conformation in contrast to open linear peptide.

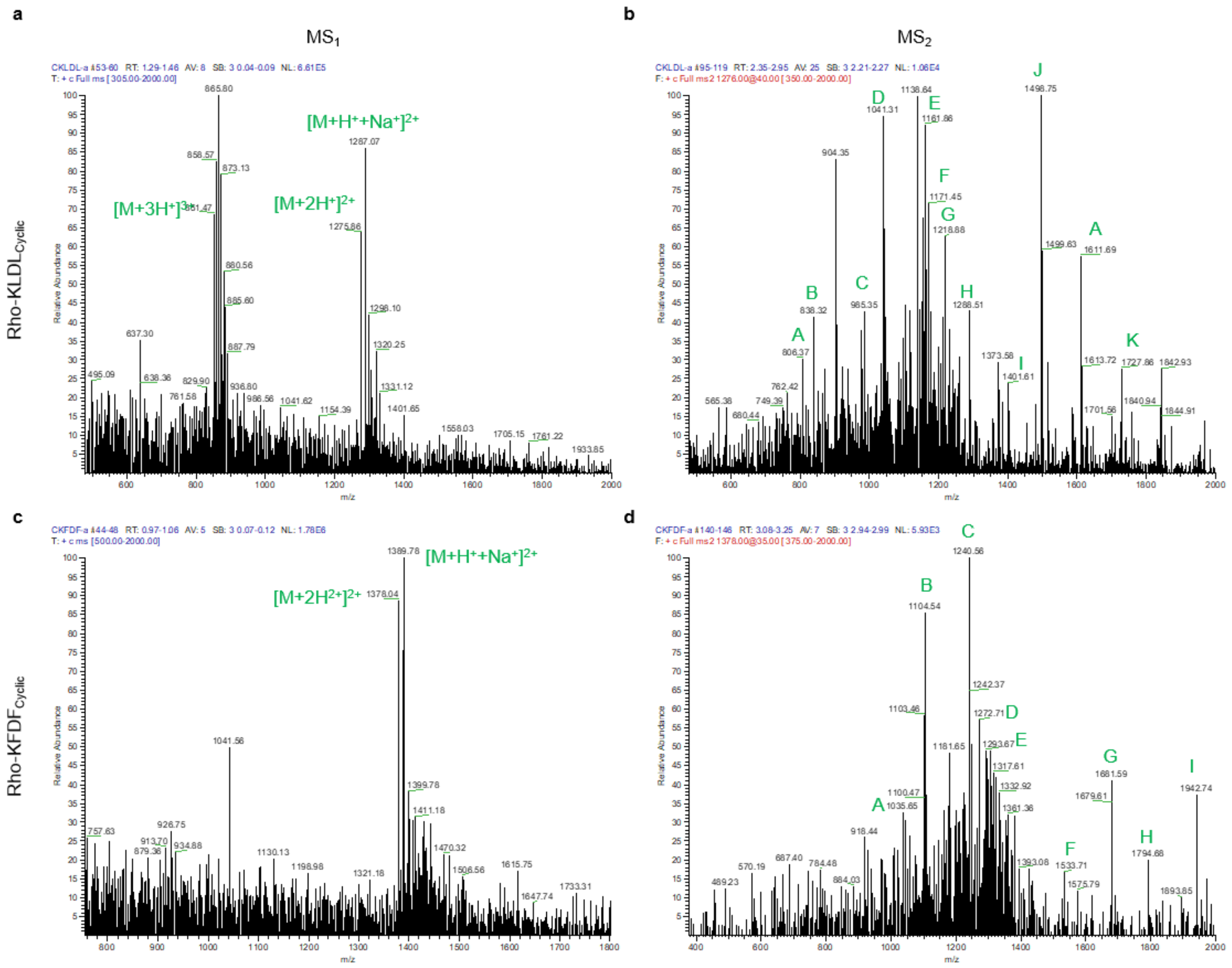

Supplementary Fig. 8.Tandem-MS of Rho-KLDL$_{Cyclic}$ and Rho-KFDF$_{Cyclic}$ progelators. Fragmentation of the peptide sequence enables identification of the peptide sequence, notably those that contain the less labile covalent disulfide bond. **a**, MS^1 spectrum for Rho-KLDL$_{Cyclic}$ with peak identities labeled in green. **b**, Corresponding MS^2 fragmentation spectrum filtered for the 1276 m/z ion. Peak identities, as determined by DisConnect software, are displayed in **Supplementary Table 3**. **c**, MS^1 spectrum for Rho-KLDL$_{Cyclic}$ with peak identities labeled in green. **d**, Corresponding MS^2 fragmentation spectrum filtered for the 1378 m/z ion. Peak identities, as determined by DisConnect software, are displayed in **Supplementary Table 4**. Note that only normal fragmentation peaks (no H_2O, NH_3, or CO losses) are identified

.

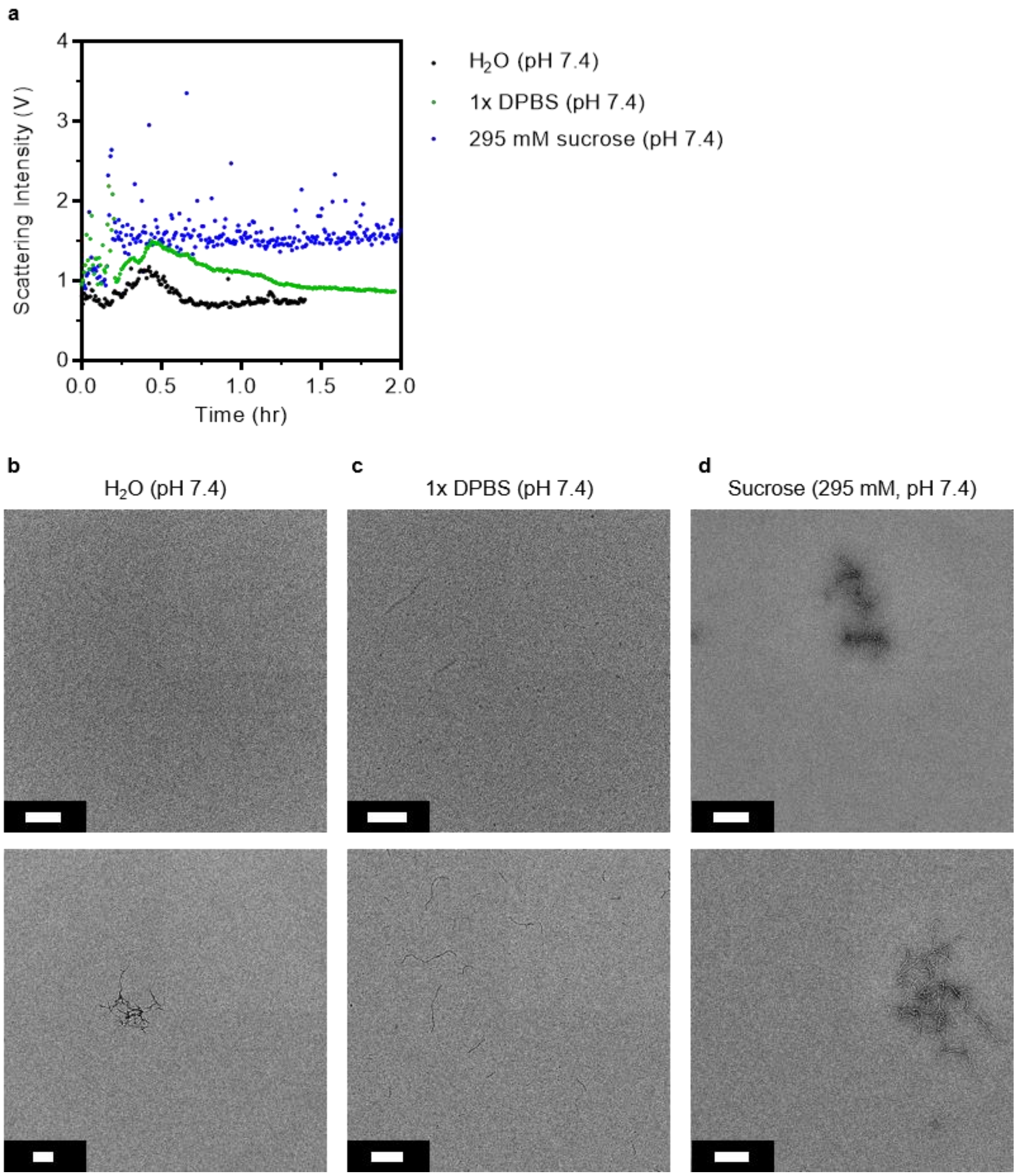

Supplementary Fig. 9. SLS profiles for unlabeled progelator in different buffers. Solutions of KFDF$_{Cyclic}$ were formulated in solvents commonly used for formulating SAPs to identify optimal solvents that minimize the occurrence of large assemblies in solution. **a**, KFDF$_{Cyclic}$ was pre-dissolved at 10 mg mL^{-1} in H_2O at pH 9 and added to solvent in the DLS cuvette such that final concentrations were 1 mg mL^{-1} in H_2O, 1x DPBS, or 295 mM sucrose (pH 7.4). Scattering was highest for sucrose solutions and equivalent in H_2O and 1x DPBS after 2 hr incubation indicating smaller assemblies in the later solvent. Intensities were corrected for background solvent scattering. Scattering intensities were normalized to individual solvents. **b-d**, TEM analysis in H_2O (**b**), 1x DPBS (**c**), and 295 mM sucrose (**d**), diluted to 100 µM. Large structures were absent in H_2O and 1x DPBS with some small clusters of fibrils in sucrose. Top row scale bar 100 nm. Bottom row scale bar 200 nm.

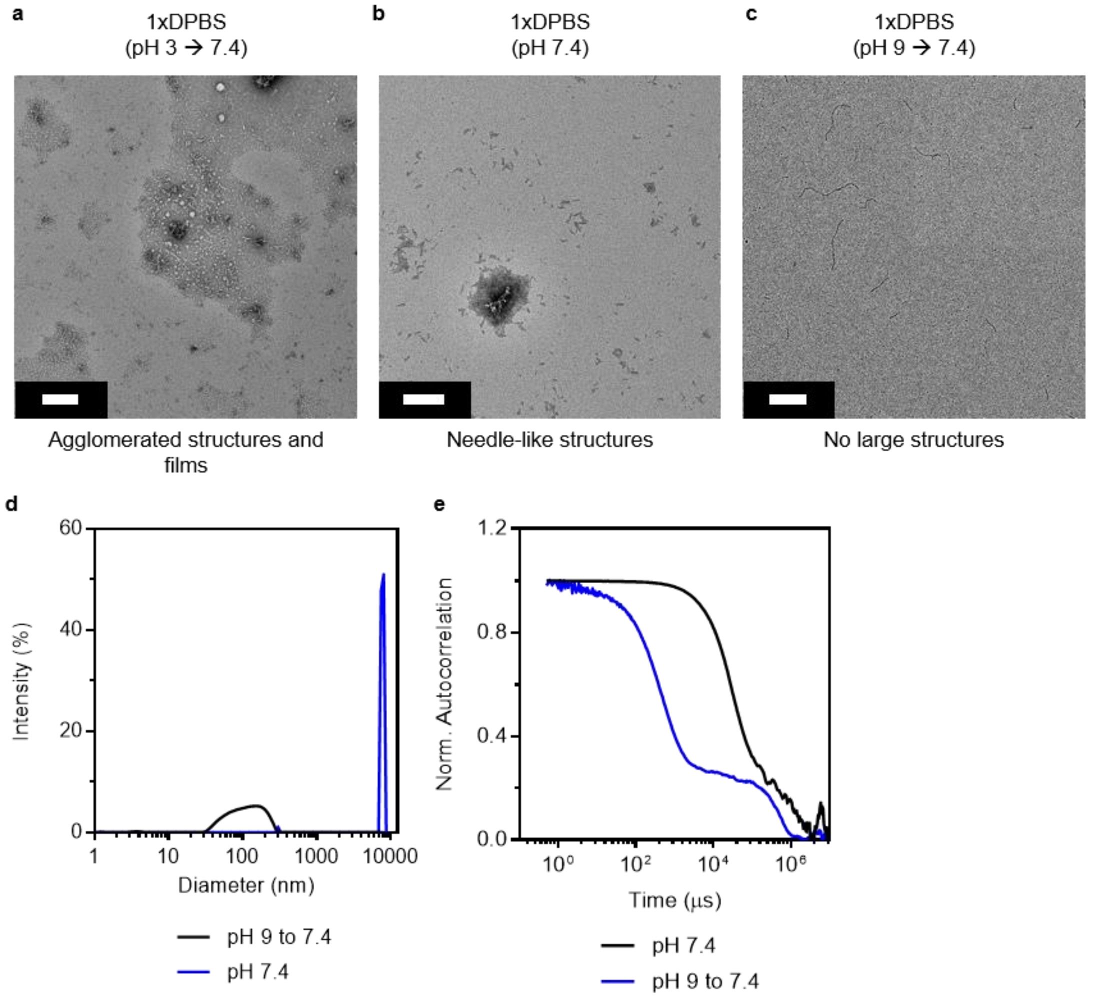

Supplementary Fig. 10. Effect of cyclic peptide progelator formulation on aggregation. Solutions of $KFDF_{Cyclic}$ were optimally formulated in 1x DPBS using a pH switch from basic (pH 9.0) to neutral (pH 7.4) conditions prior to addition of saline to limit aggregation. These formulations in 1x DPBS are stable over prolonged incubation periods (up to 3 days) under dilute (100 µM) and concentrated (10 mM) conditions. **a-c**, TEM (100 µM) micrographs of progelator formulated in 1x DPBS after solvent switch from (**a**) acidic to neutral pH, (**b**) no pH switch, or (**c**) switch from basic to neutral pH. Scale bar 200 nm. d-e, DLS (1 mg mL^{-1}) (**d**) and the corresponding normalized autocorrelation functions (**e**) depict the influence of different formulation conditions on aggregation.

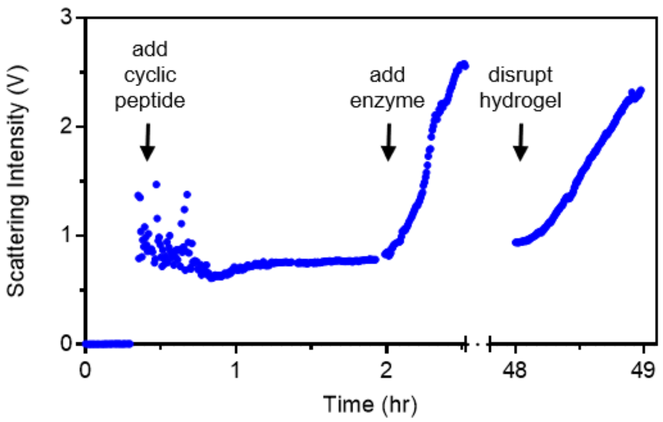

Supplementary Fig. 11. SLS of cyclic progelator before and after enzyme addition. This scattering profile demonstrates that progelator in solution is stable to flocculation for hours until addition of thermolysin induces a steep increase in scattering intensity as self-assembly occurs. Complete disruption of macromolecular structures via sonication at 48 hr post-activation results in a return to nominal scattering intensity like that of the cyclic progelator. Reassembly is observed by a gradual increase in scattering intensity of this dilute solution. Final peptide concentration is 1 mg mL^{-1} in 1xDPBS at 37 ˚C. Hydrogel disruption was achieved with 10 min sonication.

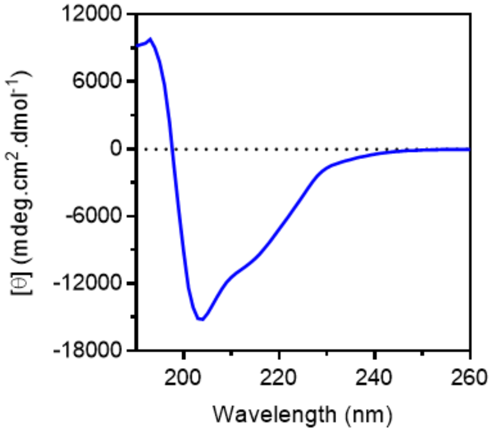

Supplementary Fig. 12. Circular dichroism (CD) of KFDF$_{\mathbf{Cyclic}}$ progelator.a, CD spectrum of KFDF$_{Cyclic}$ progelator (500 μM, pH 7.4) formulated with basic to neutral pH solvent switch. (n=3 repeats). The magnitude of the high energy minimum at 204 nm is increased significantly following cyclization of KFDF$_{Linear}$ (from **Error! Reference source not found.**b in main text), which is attributed to aromatic π-π* effects achieved through vertically stacked macrocycles

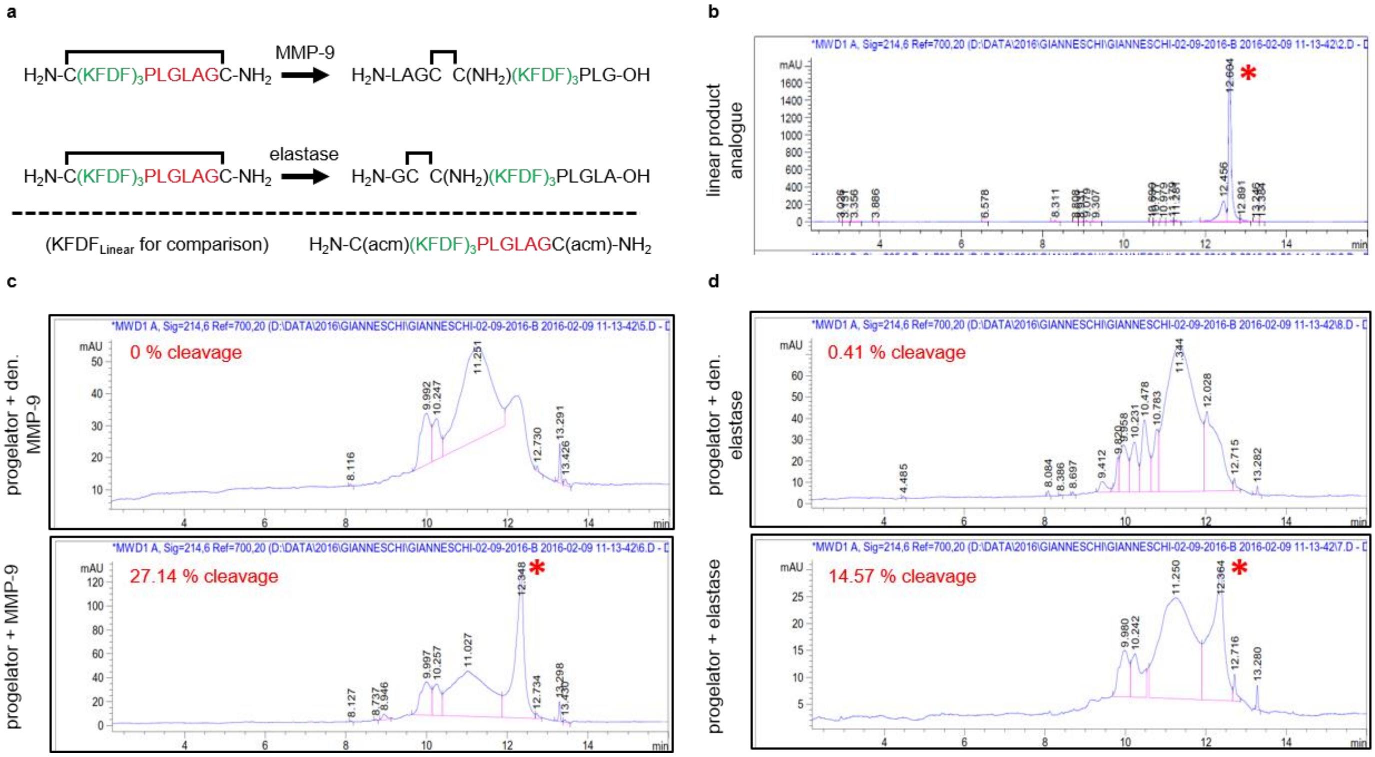

Supplementary Fig. 13. Representative enzyme cleavage of unlabeled cyclic progelator. $KFDF_{Cyclic}$ was treated with MI-associated enzymes, MMP-9 and elastase to confirm enzyme responsiveness. a, Sequences of the $KFDF_{Cyclic}$ progelator cleavage reactions and $KFDF_{Linear}$ analogue for comparison. b, LCMS spectrum of $KFDF_{Linear}$ analogue (R_t = 12.5 min) as a positive control. c, LCMS spectra of $KFDF_{Cyclic}$ progelator treated with denatured (top) and active (bottom) MMP-9 catalytic domain for 5 hr. d, LCMS spectra of $KFDF_{Cyclic}$ progelator treated with denatured (top) and active (bottom) porcine elastase for 5 hr. Calculated percent cleavages are reported in the spectra. Cleavage products are indicated with red asterixis. Due to the large number of cis/trans configurational isomers and amphiphilic self-assembling nature of our cyclic peptides, our pure $KFDF_{Cyclic}$ progelator eluted within an R_t range between 9.7-12.5 min. Cleaved peptide peaks (R_t ~ 12.4 min) have better resolution likely due to increased molecular flexibility. Cleavage reactions are described in the Methods section of the main text.

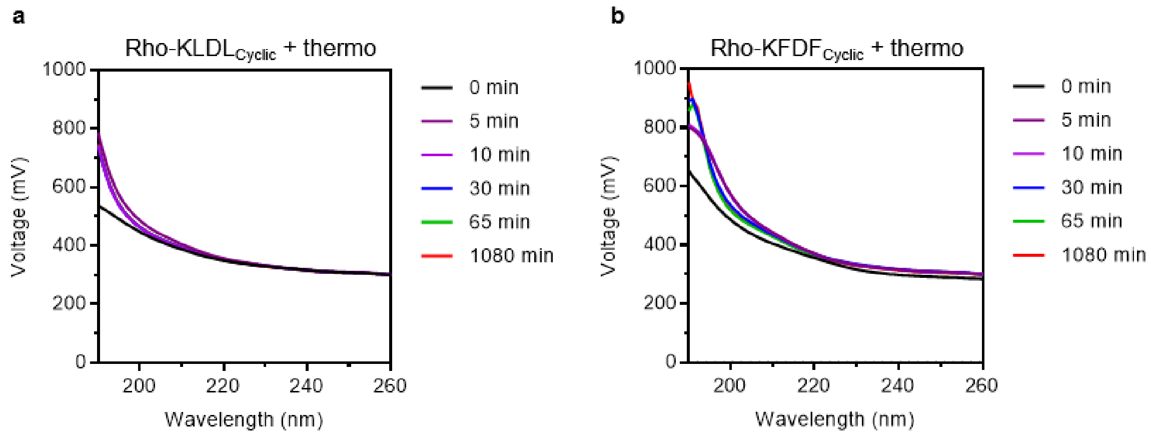

Supplementary Fig. 14. Voltage plots for thermolysin cleavage of labeled progelators by CD. Corresponding data for **Error! Reference source not found.**c,f CD spectra of time-course analysis of thermolysin cleavage. a, High tension (HT) voltage plot of thermolysin cleavage of Rho-KLDL$_{Cyclic}$ over time. b, High tension (HT) voltage plot of thermolysin cleavage of Rho-KFDF$_{Cyclic}$ over time. Detector saturation at wavelengths where HT voltage > 800 mV. Peptide concentration 125 µM. [substrate:enzyme] = 4500:1.

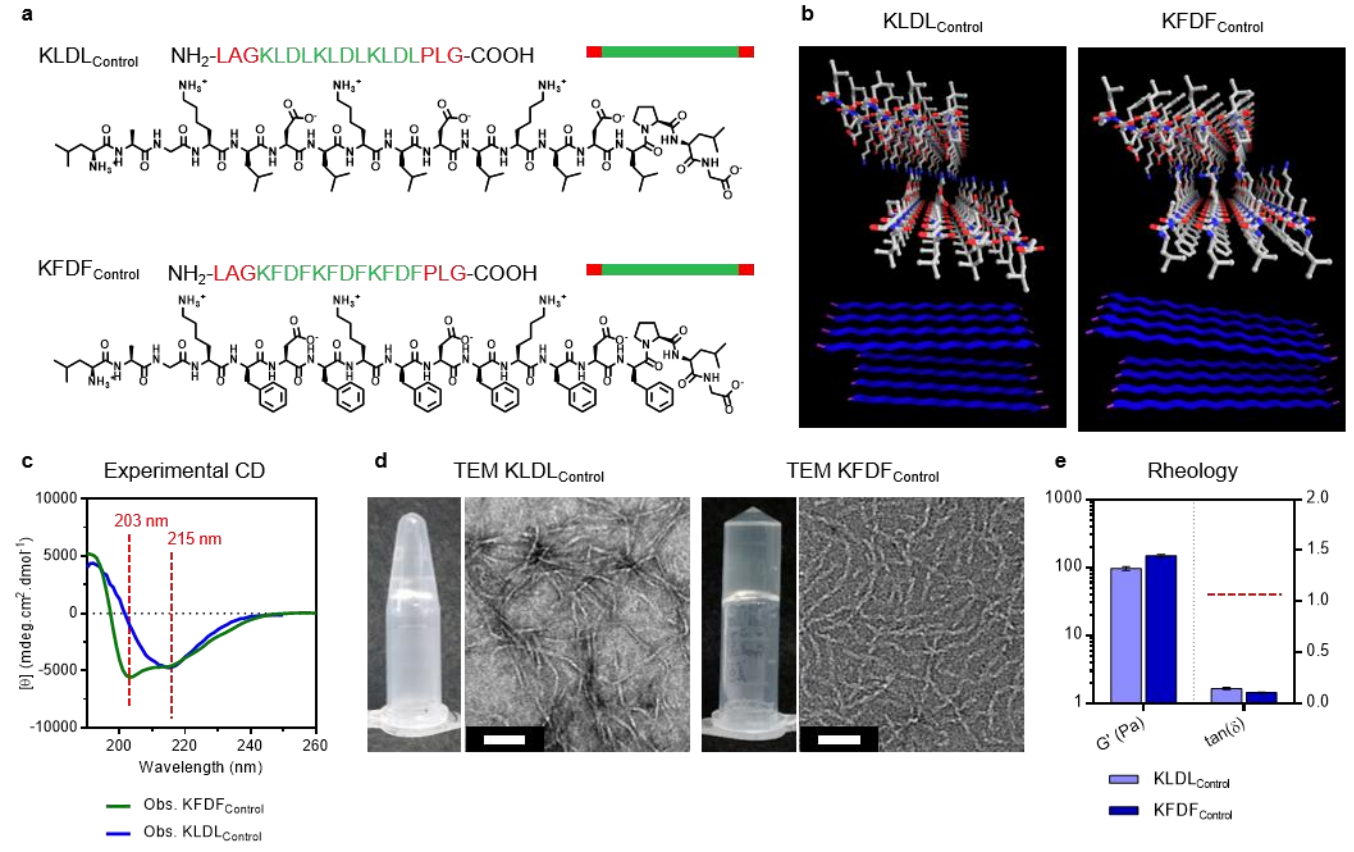

Supplementary Fig. 15. Simulated control SAPs, KLDL$_{Control}$ and KFDF$_{Control}$, for progelator cleavage. KLDL$_{Control}$ and KFDF$_{Control}$ SAPs were used to represent the enzymatically cleaved progelators containing flanking substrate residues on the gelling sequences. **a**, Sequences, chemical structures, and corresponding diagrammatical representations of peptides, with green denoting gelator sequence and red denoting cleaved substrate recognition sequences. **b**, Predicted structures and ribbon diagrams (bottom) of KLDL$_{Control}$ and KFDF$_{Control}$ SAPs from FibPredictor simulations revealing parallel β-sheet orientations (see **Supplementary Table 1-2**). **c**, Experimental circular dichroism (CD) spectra of KLDL$_{Control}$ and KFDF$_{Control}$ SAPs, with labeled minima at 215 nm ($n \rightarrow \pi^*$) and 203 nm ($\pi \rightarrow \pi^*$). **d**, Assembled hydrogel photographs and corresponding TEM images of KLDL$_{Control}$ (left) and KFDF$_{Control}$ (right) SAPs. Scale bar 50 nm. **e**, Viscoelastic measurements of storage moduli, G' (Pa), and damping factor, tanδ, for KLDL$_{Control}$ and KFDF$_{Control}$. Gel capacity is defined as tanδ < 1 (dashed line). CD measurements performed at 500 µM SAP in 10 mM Tris buffer, pH 7.4. (n=3 repeats) SAPs for TEM and rheology prepared at 100 µM and 15 mg mL^{-1} in 1xDPBS (pH 7.4), respectively. Rheological measurements reported for angular frequency of 2.5 rad s^{-1}. (n=3 repeats) Values are mean ± SEM.

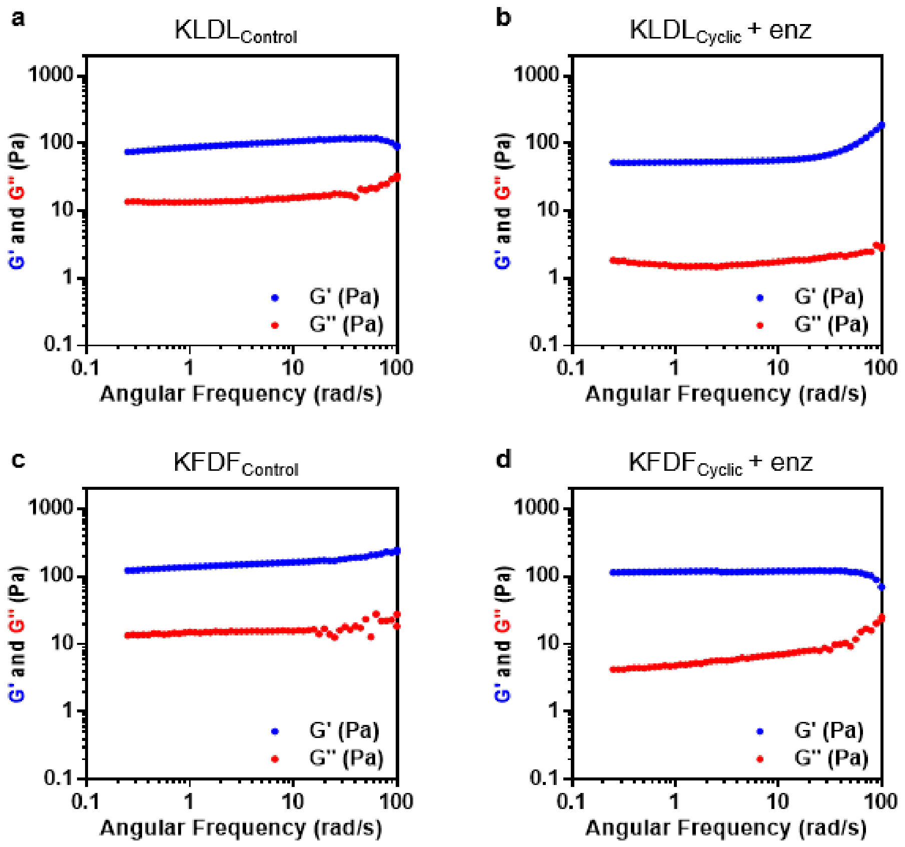

Supplementary Fig. 16. Frequency sweeps of product analogues and cleavage products. Product analogues ($KLDL_{Control}$ and $KFDF_{Control}$) and enzymatically cleavage progelators ($KLDL_{Cyclic}$ + enz and $KFDF_{Cyclic}$ + enz) were analyzed for frequency dependence of the viscoelastic moduli across low and high frequency. **a-b**, Frequency sweeps for (**a**) $KLDL_{Control}$ and (**b**) $KLDL_{Cyclic}$ + enz hydrogels. **c-d**, Frequency sweeps for (**a**) $KFDF_{Control}$ and (**b**) $KFDF_{Cyclic}$ + enz hydrogels. Hydrogels at 10 mM in 1xDPBS (pH 7.4). Angular frequency sweeps (100-0.25 rad s^{-1}) at 0.5% strain (n=3 repeats). Values are mean ± SEM.

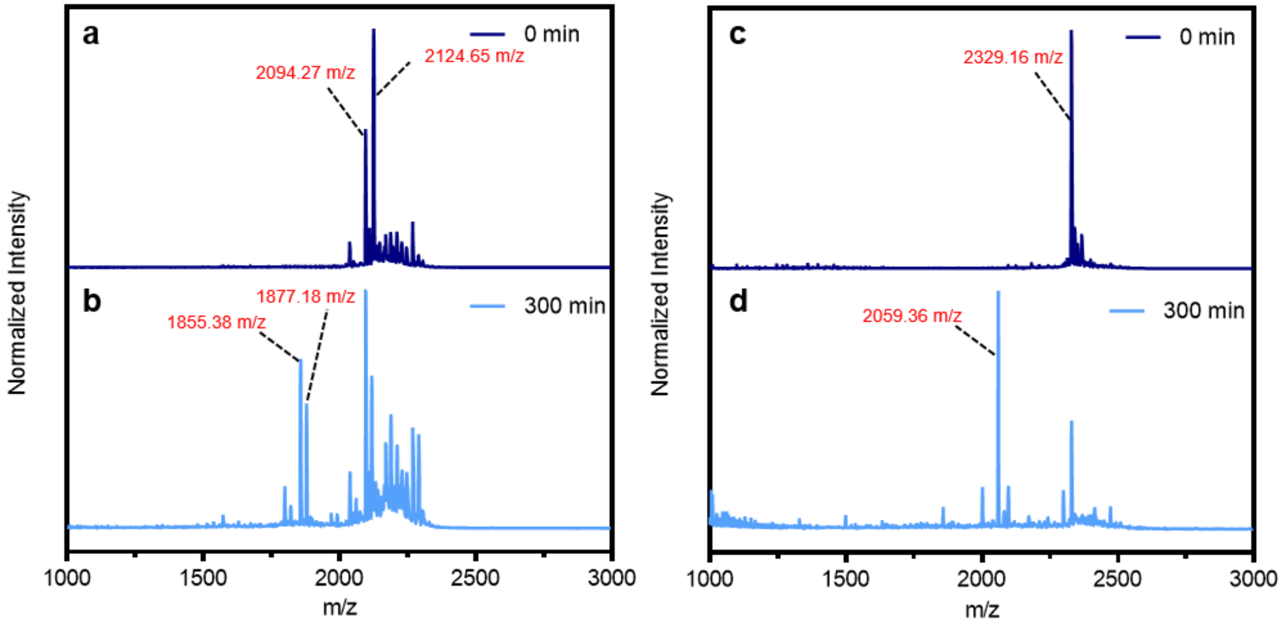

Supplementary Fig. 17. MALDI spectra of KLDL$_{Cyclic}$ and KFDF$_{Cyclic}$ treated with thermolysin. a-d, Peptides (500 µM in H_2O) treated with thermolysin (1:4500 enzyme/substrate molar ratio) for 300 min. **a**, Masses for KLDL$_{Cyclic}$ at 2124.65 m/z and 2094.27 m/z correspond to [M-16+H$^+$], and [M-S-16+H$^+$], respectively. Deamination as a result of N→S acyl transfer at the C-terminal cysteine amide is suspected to cause the Δ-16 mass difference observed by MALDI for both cyclic progelators. **b**, Masses for KLDL$_{Cyclic}$+thermolysin are 1855.38 m/z and 1877.18 m/z correspond to [M+H$^+$] and [M+Na$^+$] for linear SAP after loss of Leu-Gly-Leu from h$_2$n-*CKLDLKLDLKLDLP/LGL/AGC*conh$_2$ (* is disulfide bond; / indicates cut sites), as indicated in main text **Figure 5a**. **c**, Mass for KFDF$_{Cyclic}$ at 2329.16 m/z corresponds to [M-16+H$^+$]. Deamination as a result of N→S acyl transfer at the C-terminal cysteine amide is suspected to cause the Δ-16 mass difference observed by MALDI. **d**, Mass for KFDF$_{Cyclic}$+thermolysin is 2059.36 m/z, corresponds to [M+H$^+$] for linear SAP after loss of Leu-Gly-Leu from h$_2$n-*CKFDFKFDFKFDF/LGL/AGC*conh$_2$ (* is disulfide bond; / indicates cut sites), as indicated in main text **Figure 5d**. Lack of buffering salts to improve MALDI signal likely slows enzymatic cleavage kinetics.

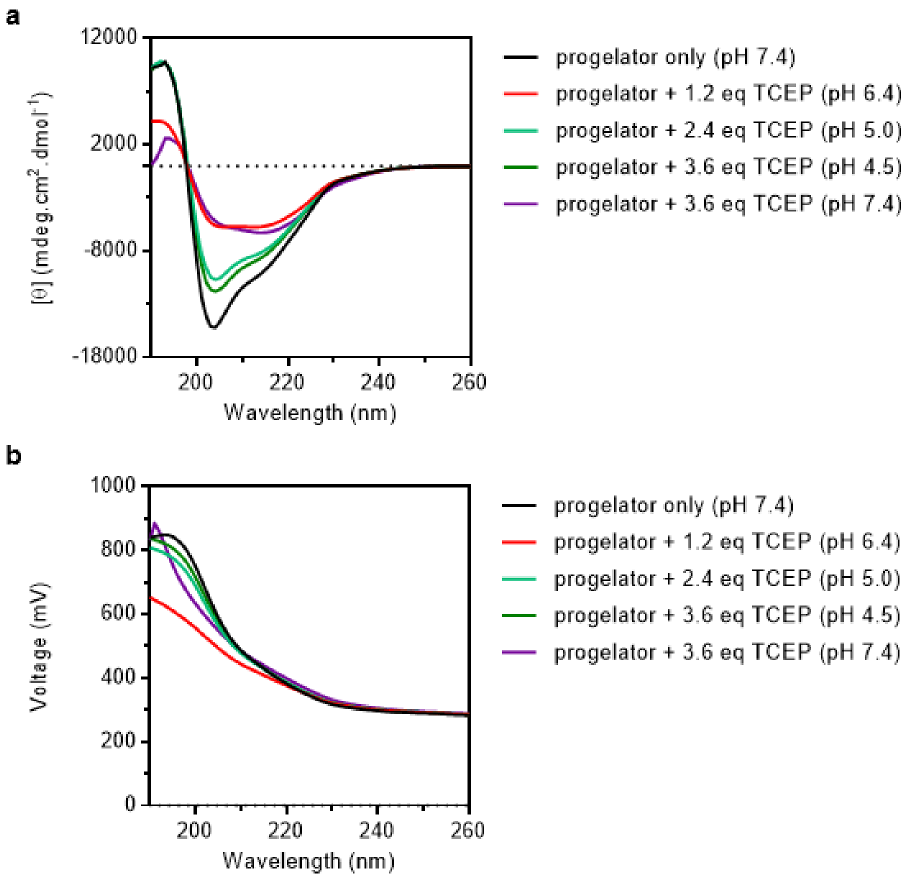

Supplementary Fig. 18. TCEP reduction of progelator. TCEP reduction indicates ideal assemblies from when cleavage of cyclic progelators is complete. TCEP reduction is shown to be reversible through TCEP inactivation at lower pH a, Progelator treated with 1.2, 2.4, and 3.6 eq of TCEP. Gradual decrease in pH (pH 7.4 to 4.5) occurs from successive TCEP additions. Neutralization to pH 7.4 with NaOH revives TCEP reductive potential. b, Corresponding high tension (HT) voltage plots. Concentration 500 µM progelator. (n=3 repeats).

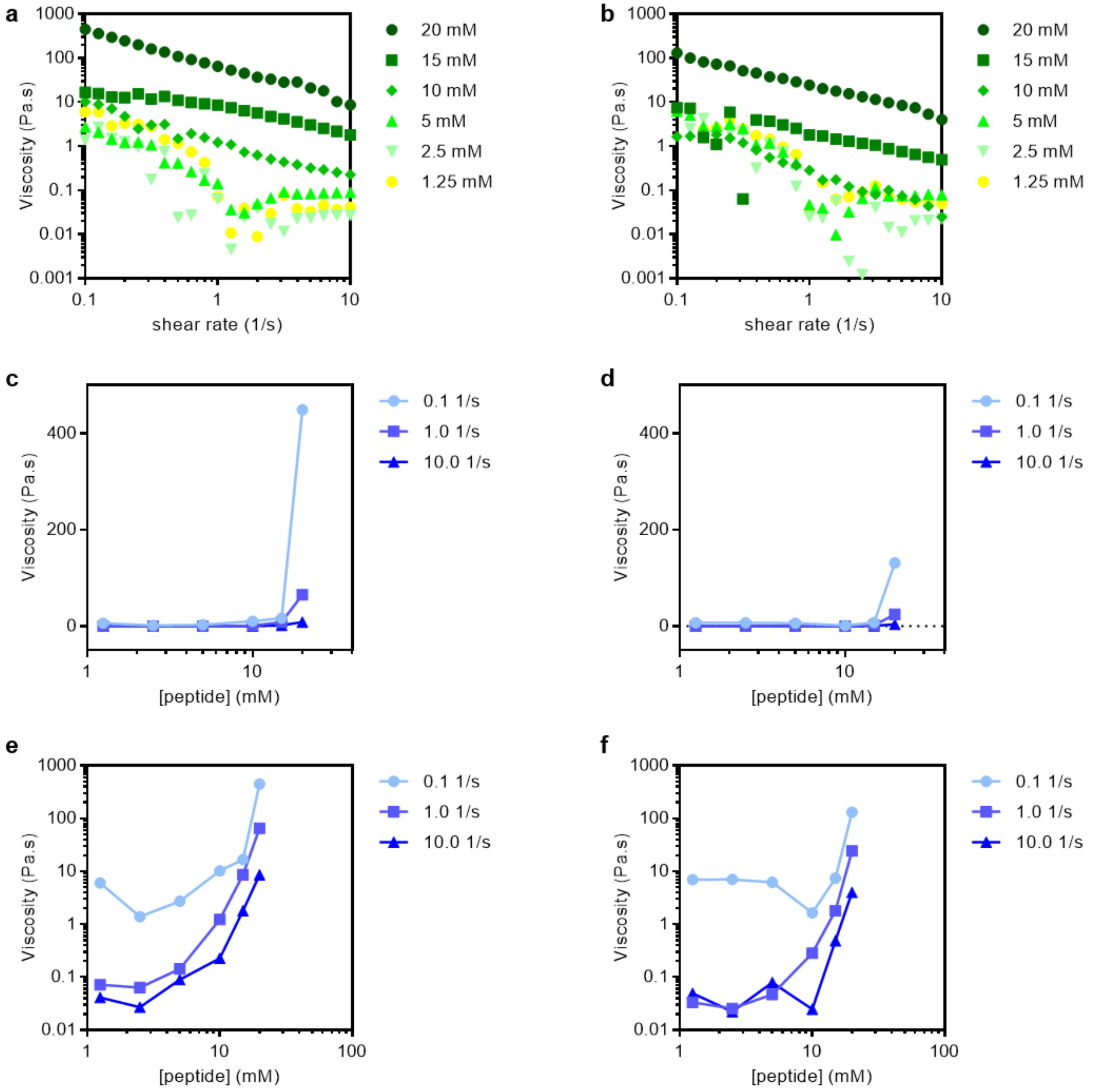

Supplementary Fig. 19. Viscosity dependence of cyclic progelators on peptide concentration. a-b, Complex viscosity of (a) KLDL$_{Cyclic}$ and (**b**) KFDF$_{Cyclic}$ as a function of peptide concentration at 1.25, 2.5, 5, 10, 15, and 20 mM. **c-f**, Viscosity is plotted with the y-axis as linear (**c-d**) or log (**e-f**) scale for both (**c, e**) KLDL$_{Cyclic}$ and (**d, f**) KFDF$_{Cyclic}$ as measured at shear rates of 0.1, 1.0, and 10.0 s^{-1}. Viscosity is primarily independent of concentration up to 10 mM. At 20 mM, viscosity increases at lower shear rates and exits the regime of catheter injectability; however, it remains unaffected at the highest shear rate of 10 s^{-1}, corresponding to shear-thinning behavior during injection.

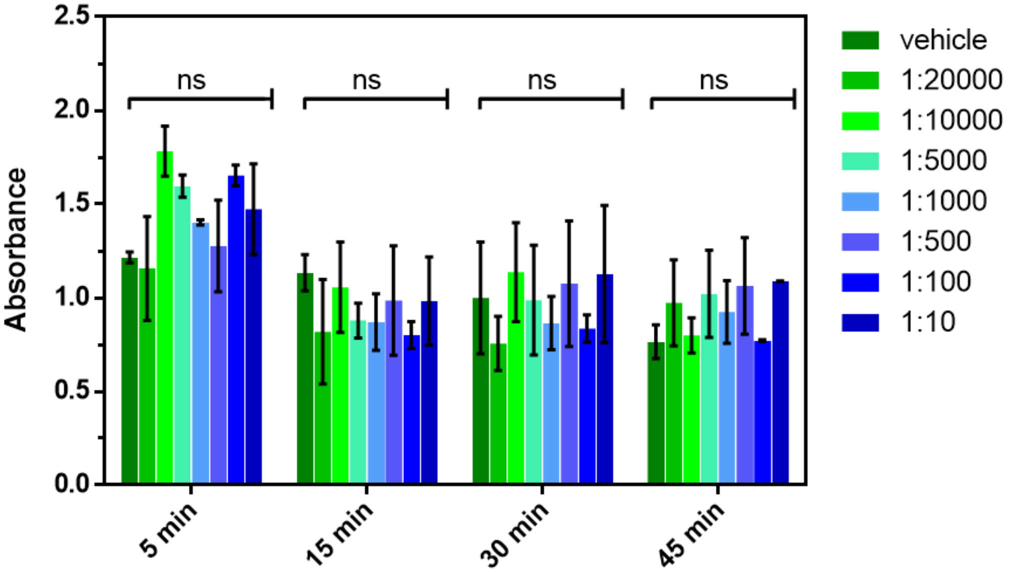

Supplementary Fig. 20. Hemostasis kinetics as a response of dosing. Supplementary data corresponding to Error! Reference source not found.**d** in main text. Extent of clotting measured as absorbance at 405 nm as a function of dosing at 5, 15, 30, and 45 min in whole human blood. ns ($p > 0.05$). ($n=4$ per group). Two-way ANOVA at each timepoint for comparison with vehicle standard. Values are mean ± SEM.

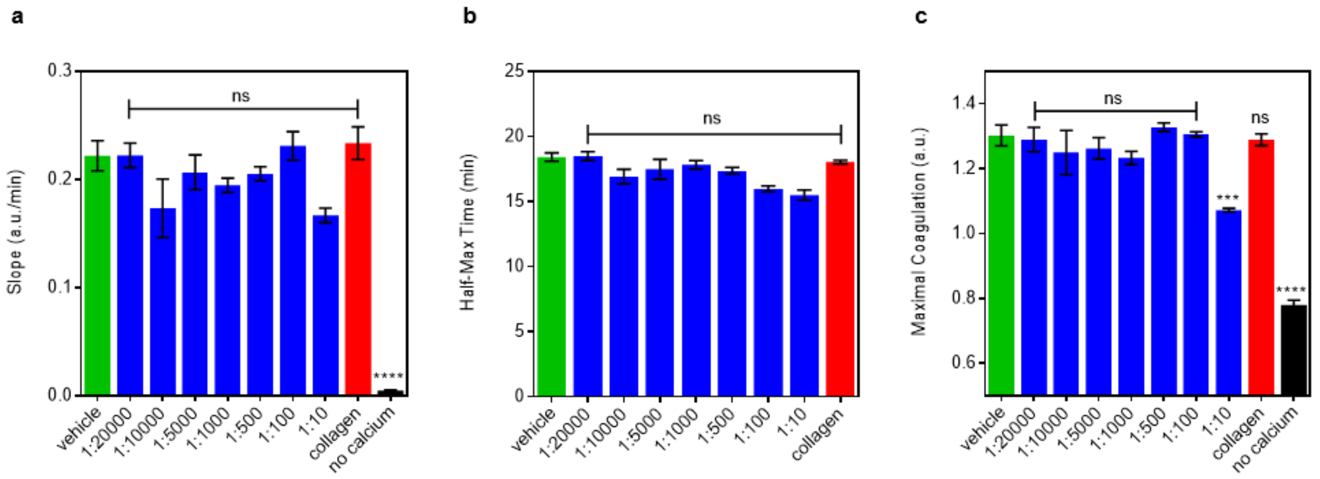

Supplementary Fig. 21. Pro-thrombotic assay statistics. Supplementary data corresponding to Error! Reference source not found.**f** in main text. **a**, Coagulation rate measured as the maximal first derivative, or slope, of coagulation. **b**, Time at which coagulation is at half maximal completion. **c**, Extent of coagulation measured as the plateau absorbance. (n=6 group). Progelator concentrations are 1:20000, 1:10000, 1:5000, 1:1000, 1:500, 1:100, and 1:10 volume dilution in PPP. ns ($p > 0.05$), *** ($p \leq 0.001$), **** ($p \leq 0.0001$). Ordinary one-way ANOVA for comparison with vehicle standard. Values are mean ± SEM.

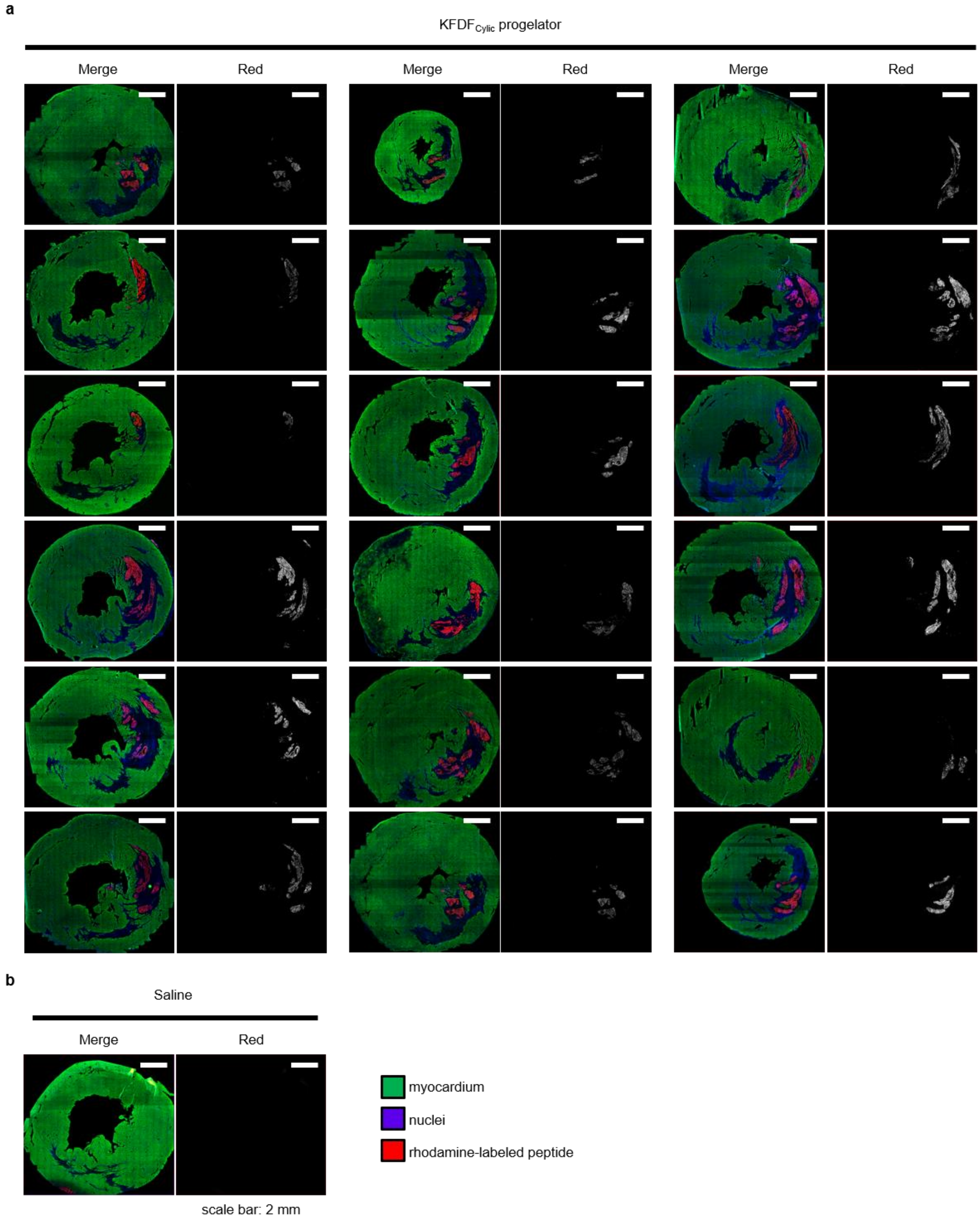

Supplementary Fig. 22. Heart slices visualized through IHC staining. a, Merged (left) and red channel-only (right) fluorescence images of 18 slices from 5 rat hearts that received intramyocardial injections of $KFDF_{Cyclic}$ progelator at 7 days

post-MI and harvested 24 hr later. Images correlate with main text Error! Reference source not found.. Tissue is stained for myocardium with anti-α-actinin antibody (green) and nuclei with Hoechst (blue), and peptides are visualized through the rhodamine tag (red). Peptide is localized within the MI region (+blue-green). **b**, Representative slice of heart receiving saline injection at 7 days post-MI and harvested 24 hr later. Scale bar 2 mm

.

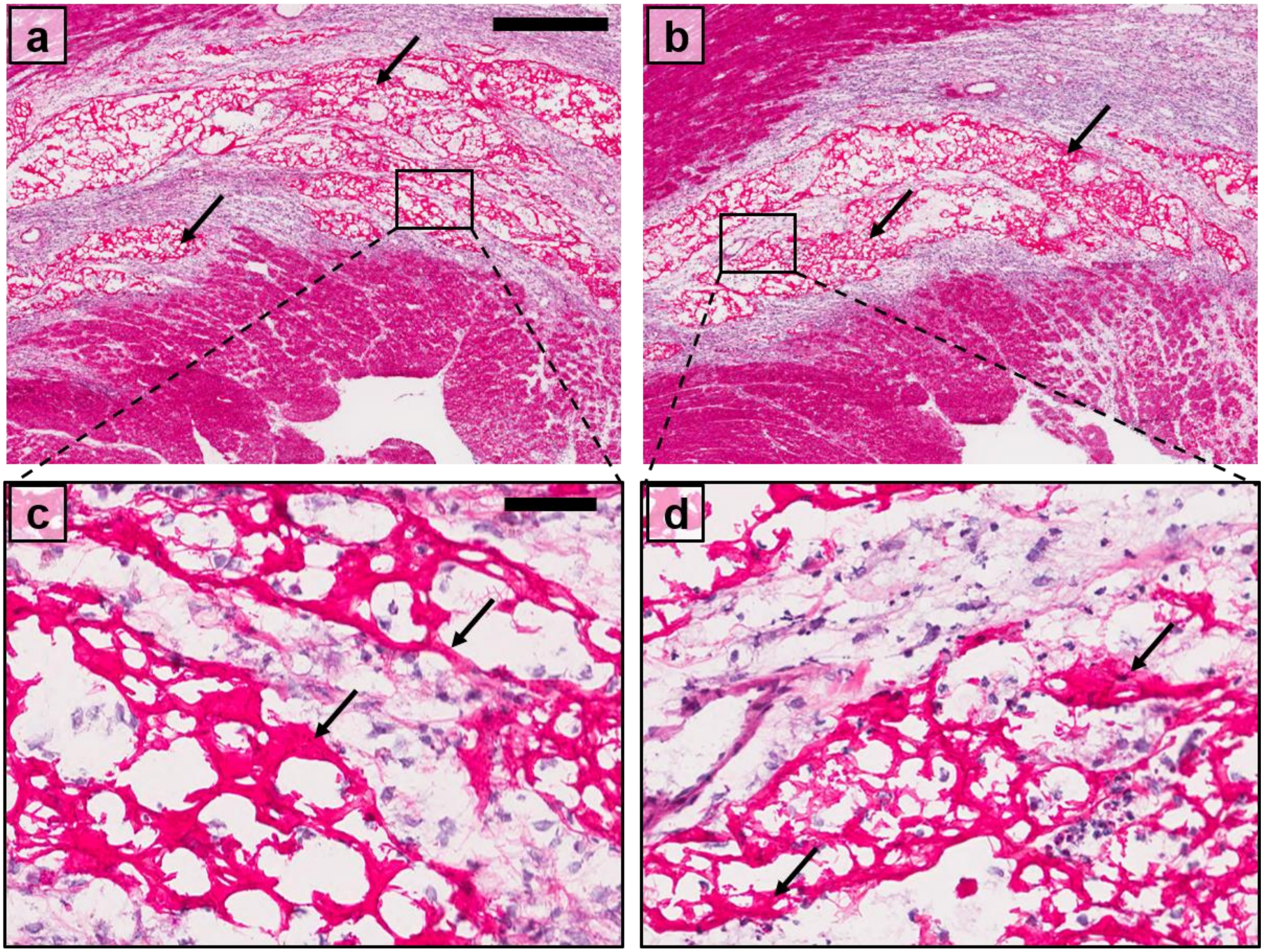

Supplementary Fig. 23. Representative H&E slices of injected heart. a-d, Intramyocardial injection of $KFDF_{Cyclic}$ progelator (75 µL, 10 mM in 1x DPBS) one day post-injection (8 days post-MI). Eosinophilic peptide from progelator injection stains bright pink as indicated by black arrows. **a-b**, Scale bar 0.5 mm. **c-d**, Scale bar 50 µm.

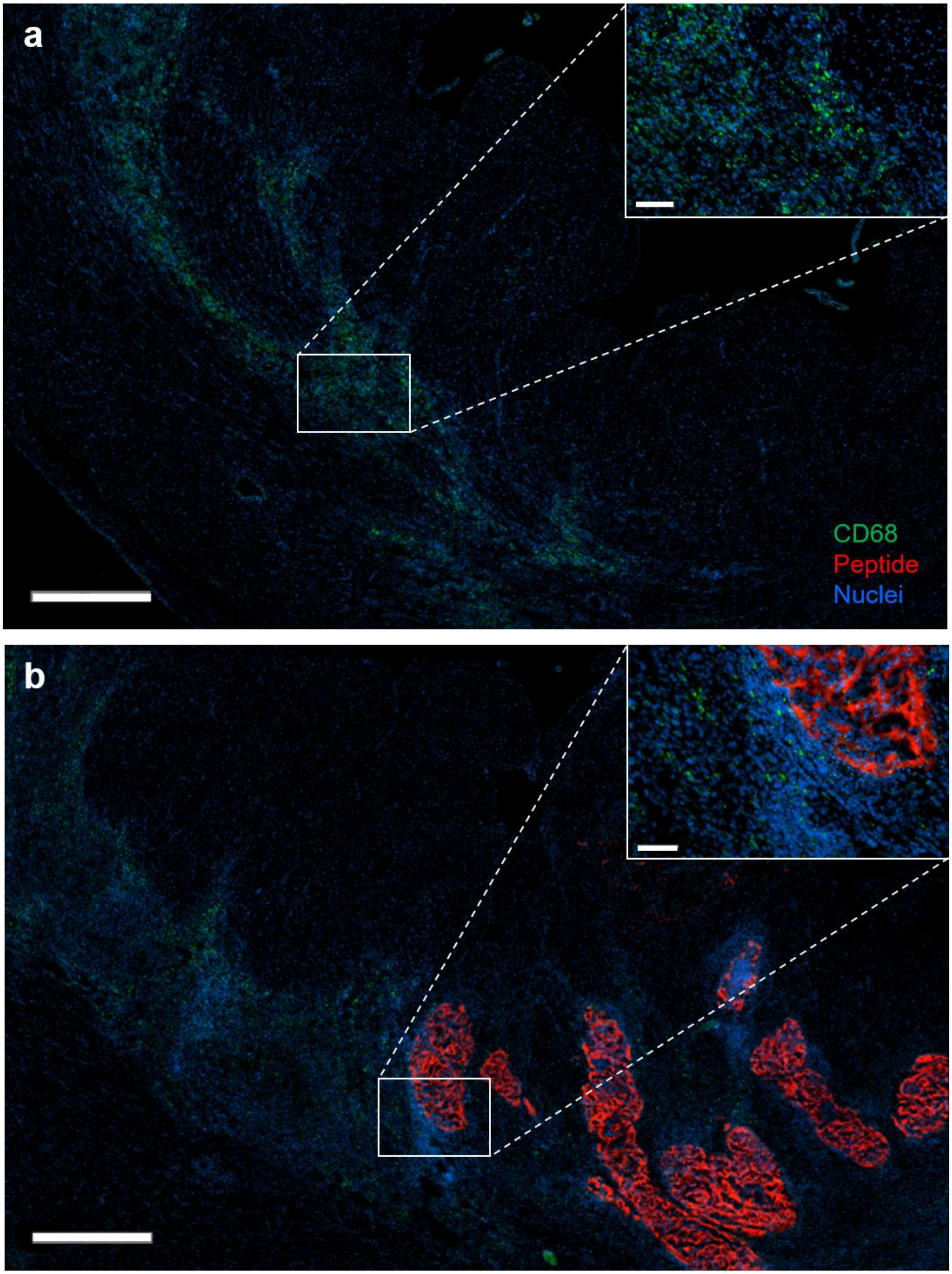

Supplementary Fig. 24. Representative tissue staining for inflammatory response. Animals received intramyocardial injection of saline (n=3) or $KFDF_{Cyclic}$ progelator (n=5) at 7 days post-MI and hearts were harvested at 24 hr post-injection. **a-b**, CD68+ staining for macrophages shows distribution throughout the infarct region, which was similar in both the (**a**) saline and (**b**) peptide injected groups. Scale bar 700 μm and 70 μm (inset).

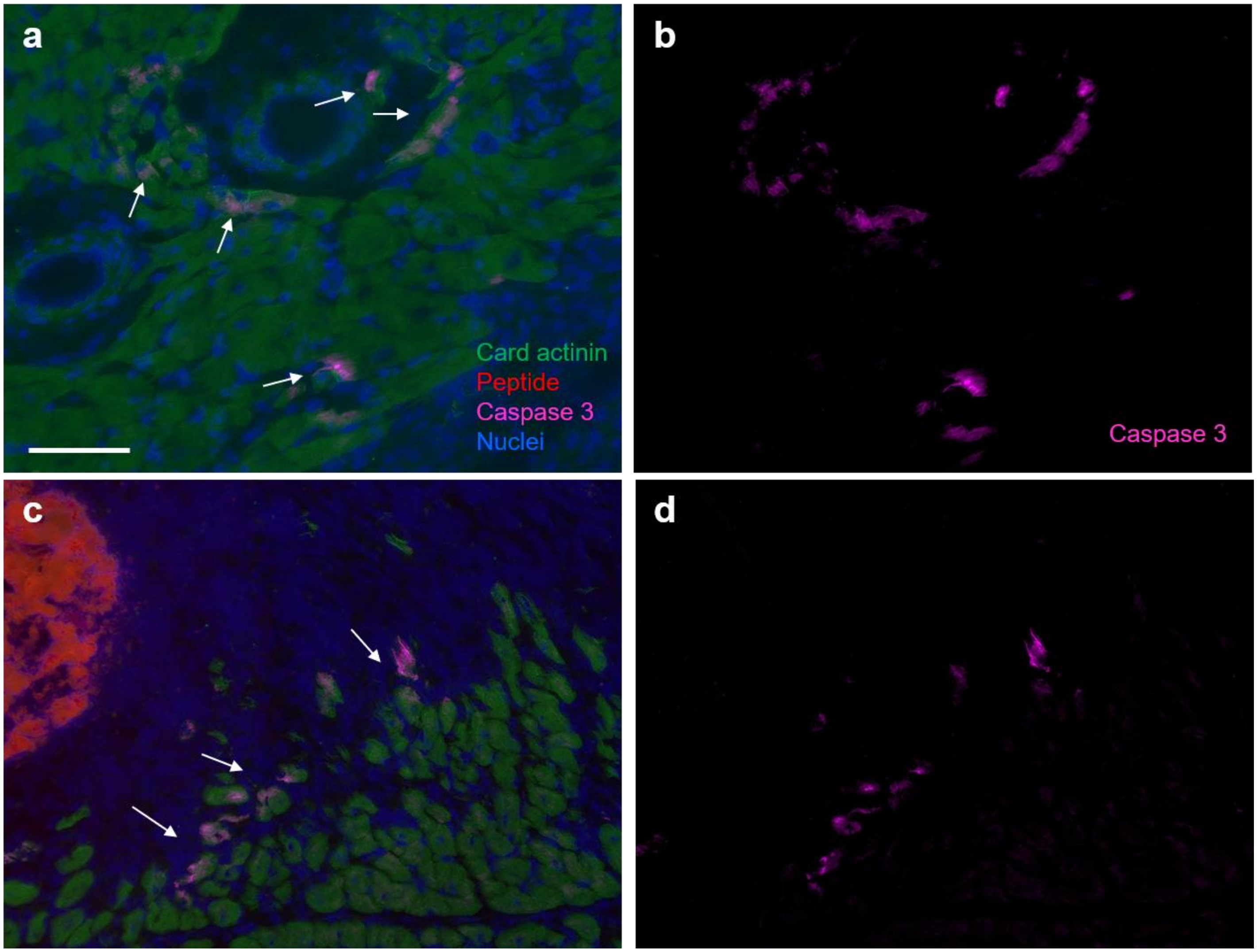

Supplementary Fig. 25. Representative tissue staining for cardiomyocyte apoptosis. Animals received intramyocardial injection of saline (n=3) or $KFDF_{Cyclic}$ progelator (n=5) at 7 days post-MI and hearts were harvested at 24 hr post-injection. Cleaved caspase-3 staining for apoptosis shows few apoptotic cells, mostly in the border zone. **a-b**, Saline injected group displaying (**a**) merge of all channels or (**b**) cleaved caspase-3 only. **c-d**, Peptide injected group displaying (**c**) merge of all channels or (**d**) cleaved caspase-3 only. Arrows indicate apoptotic cells. Scale bar 100 µm.

Supplementary Table 1. FibPredictor experimental setup over three generations. Input included the peptide sequence, number of strands per sheet (x 2 sheets total) to model, and selection criterion about scoring functions, and rotation operations performed on the second β-spine relative to the first β-spine for generating all classes of amyloid fibrils. The number of random models, and distances angle variation between sheets, were also selected to improve modeling conditions. Changes to parameters with each future generation were a result of model analysis from the (n-1) generation.

	Generation 1	Generation 2	Generation 3
Number of strands per sheet	2	4	4
Sense of β-sheet orientation	Both	Antiparallel or parallel	Selected in Gen 2
Scoring function(s)	Amb_3b_score only	Amb_3b and GOAP score	Amb_3b and GOAP score
Rotations	x,z,zx,no rotation	x,z,zx,no rotation	x,z,zx,no rotation
Random models	30	60	200
Distance variation between the sheets	7 Å	7 Å	6 Å
Minimum distance between the sheets	3 Å	3 Å	4 Å
Angle variation between the sheets	45˚	45˚	10˚

Supplementary Table 2. Experimental CD and theoretical modeling of gelators. Corresponds with Error! Reference source not found.Error! Reference source not found. in main text. Observed shift in high energy minimum in CD spectra indicate reorientation in 2˚ structure. Calculated orientations for minimal total energy structures generated in FibPredictor agree with observed inferences. Internal scoring SCWRL4 energies and corresponding empirical (Amber_3b) and statistical (SCWRL4) scores for minimal energy models are displayed for the top model of each sequence. Normalized GOAP scores are provided for comparison of fibrils of different sizes. Scores are unitless.

Peptide	Sequence	Obs. CD $\pi \rightarrow \pi^*$ Peak	Obs. CD Inference	Theor. CD Orientation	SCWRL4 energy	Amb_3b score	GOAP score	GOAP normalized
KLDL	(KLDL)$_3$	---	antiparallel	antiparallel	353.796	-96.984	-7271.93	-75.7493
KLDL$_{Control}$	LAG(KLDL)$_3$PLG	Blue-shifted	Parallel	Parallel	351.78	-112.363	-7675.04	-53.2989
KLDL$_{Linear}$	C(KLDL)$_3$PLGLAGC	Blue-shifted	Parallel	Parallel	370.407	-128.576	-8610.19	-53.8137
KFDF	(KFDF)$_3$	----	Antiparallel	Antiparallel	621.622	-53.771	-7179.08	-74.7821
KFDF$_{Control}$	LAG(KFDF)$_3$PLG	Blue-shifted	Parallel	Parallel	262.448	-158.008	-7755.24	-53.8558
KFDF$_{Linear}$	C(KFDF)$_3$PLGLAGC	Blue-shifted	parallel	parallel	340.488	-158.782	-11096.7	-69.3543

Supplementary Table 3. DisConnect analysis of Rho-KLDL$_{Cyclic}$ progelator. Data from DisConnect software displayed in tables with peak labels corresponding to **Supplementary Fig. 8**, MS^2 fragmentation sequences, mass and charge values, and conclusions about cysteine connectivity. B is Rhodamine (413.15 m/z), | indicates separate peptides linked through a cysteine disulfide bond.

Peak	Sequence	m/z	Z (+)	Conclusion
A	BC1 | KLDLPLGLAGC2	806.36 / 1612.72	1 and 2	All Cys connected
A	BC1K | LDLPLGLAGC2	806.36 / 1612.72	1 and 2	All Cys connected
A	BC1KL | DLPLGLAGC2	806.36 / 1612.72	1 and 2	All Cys connected
A	BC1KLD | LPLGLAGC2	806.36 / 1612.72	1 and 2	All Cys connected
A	BC1KLDL | PLGLAGC2	806.36 / 1612.72	1 and 2	All Cys connected
B	DLKLDLKLDLPLGLAG	837.98	2	0|0|0|0
B	KLDLKLDLKLDLPLG	837.995	2	0|0|0|0
C	BC1K | DLKLDLPLGLAGC2	984.46	2	All Cys connected
C	BC1KLD | KLDLPLGLAGC2	984.46	2	All Cys connected
C	BC1KLDLK | DLPLGLAGC2	984.46	2	All Cys connected
C	BC1KLDLKLD | PLGLAGC2	984.46	2	All Cys connected
D	BC1 | KLDLKLDLPLGLAGC2	1041	2	All Cys connected
D	BC1K | LDLKLDLPLGLAGC2	1041	2	All Cys connected
D	BC1KL | DLKLDLPLGLAGC2	1041	2	All Cys connected
D	BC1KLD | LKLDLPLGLAGC2	1041	2	All Cys connected
D	BC1KLDL | KLDLPLGLAGC2	1041	2	All Cys connected
D	BC1KLDLK | LDLPLGLAGC2	1041	2	All Cys connected
D	BC1KLDLKL | DLPLGLAGC2	1041	2	All Cys connected
D	BC1KLDLKLD | LPLGLAGC2	1041	2	All Cys connected
D	BC1KLDLKLDL | PLGLAGC2	1041	2	All Cys connected
E	BC1K | LKLDLKLDLPLGLAGC2	1161.59	2	All Cys connected
E	BC1KL | KLDLKLDLPLGLAGC2	1161.59	2	All Cys connected
E	BC1KLDLK | LKLDLPLGLAGC2	1161.59	2	All Cys connected
E	BC1KLDLKL | KLDLPLGLAGC2	1161.59	2	All Cys connected
E	BC1KLDLKLDLK | LPLGLAGC2	1161.59	2	All Cys connected
E	BC1KLDLKLDLKL | PLGLAGC2	1161.59	2	All Cys connected
F	BC1KLDLKLDLKLD | LGLAGC2	1170.58	2	All Cys connected
F	BC1KLDLKLDLKLDL | GLAGC2	1170.58	2	All Cys connected
G	BC1KL | LKLDLKLDLPLGLAGC2	1218.13	2	All Cys connected
G	BC1KLDLKL | LKLDLPLGLAGC2	1218.13	2	All Cys connected
G	BC1KLDLKLDLKL | LPLGLAGC2	1218.13	2	All Cys connected
H	BC1KL | LGLAGC2	1287.56	1	All Cys connected
H	BC1KLD | GLAGC2	1289.51	1	All Cys connected
H	BC1KLDLK | GC2	1289.54	1	All Cys connected
I	BC1KLD | LGLAGC2	1402.59	1	All Cys connected
I	BC1KLDL | GLAGC2	1402.59	1	All Cys connected
I	BC1KLDLKL | GC2	1402.62	1	All Cys connected
J	BC1KL | LPLGLAGC2	1497.69	1	All Cys connected
J	BC1K | DLPLGLAGC2	1499.64	1	All Cys connected
J	BC1KLD | PLGLAGC2	1499.64	1	All Cys connected
K	BC1KLD | DLPLGLAGC2	1727.75	1	All Cys connected

Supplementary Table 4. DisConnect analysis of Rho-KFDF$_{\mathbf{Cyclic}}$ progelator.. Data from DisConnect software displayed in tables with peak labels corresponding to **Supplementary Fig. 8**, MS^2 fragmentation sequences, mass and charge values**,** and conclusions about cysteine connectivity. B is Rhodamine (413.15 m/z), | indicates separate peptides linked through a cysteine disulfide bond

Peak	Sequence	m/z	Z (+)	Conclusion
A	BC1K | DFKFDFPLGLAGC2	1035.445	2	All Cys connected
A	BC1KFD | KFDFPLGLAGC2	1035.445	2	All Cys connected
A	BC1KFDFK | DFPLGLAGC2	1035.445	2	All Cys connected
A	BC1KFDFKFD | PLGLAGC2	1035.445	2	All Cys connected
B	KFDFKFDFKFDFPLGLAGC2	1104.035	2	69|0|0|0
C	BC1 | DFKFDFKFDFPLGLAGC2	1240.03	2	All Cys connected
C	BC1KFD | FDFKFDFPLGLAGC2	1240.03	2	All Cys connected
C	BC1KFDF | DFKFDFPLGLAGC2	1240.03	2	All Cys connected
C	BC1KFDFKFD | FDFPLGLAGC2	1240.03	2	All Cys connected
C	BC1KFDFKFDF | DFPLGLAGC2	1240.03	2	All Cys connected
D	BC1KFDFKFDFKFDF | GLAGC2	1272.545	2	All Cys connected
E	BC1KFDFKFDFKFDFP | LAGC2	1292.56	2	All Cys connected
E	BC1KFDFKFDFKFDFPL | AGC2	1292.56	2	All Cys connected
F	BC1K | DFPLGLAGC2	1533.63	1	All Cys connected
F	BC1KFD | PLGLAGC2	1533.63	1	All Cys connected
G	BC1 | KFDFPLGLAGC2	1680.7	1	All Cys connected
G	BC1K | FDFPLGLAGC2	1680.7	1	All Cys connected
G	BC1KF | DFPLGLAGC2	1680.7	1	All Cys connected
G	BC1KFD | FPLGLAGC2	1680.7	1	All Cys connected
G	BC1KFDF | PLGLAGC2	1680.7	1	All Cys connected
H	BC1KFD | DFPLGLAGC2	1795.73	1	All Cys connected
I	BC1 | DFKFDFPLGLAGC2	1942.8	1	All Cys connected
I	BC1KFD | FDFPLGLAGC2	1942.8	1	All Cys connected
I	BC1KFDF | DFPLGLAGC2	1942.8	1	All Cys connected

Supplementary Table 5. Activated clotting times (ACT) in human blood. Supplemental data corresponding to Error! Reference source not found.**c** in main text. Average times reported ± standard error of mean (n=6 per group). Ordinary one-way ANOVA for comparison with vehicle standard. Values are mean ± SEM.

	standard vehicle	peptide in blood dilutions 1:20000	1:10000	1:5000	1:1000	1:500	1:100	1:10	positive control collagen	negative control no calcium
Average Time (sec)	178	178	174	175	173	181	184	176	150	>1500
SEM	(± 6)	(± 3)	(± 4)	(± 6)	(± 5)	(± 5)	(±2)	(±3)	(± 3)	n/a
P Value		> 0.9999	0.9941	0.9969	0.9787	0.9975	0.8982	0.9997	0.0002	< 0.0001
Summary		ns	ns	ns	ns	ns	ns	ns	***	****

Supplementary Table 6. Hemolysis of red blood cells (RBCs). Supplemental data corresponding to Error! Reference source not found.**d** in the main text. Nonhemolytic biomaterials classified by < 5% hemolysis. (n=4 per group). ns ($p > 0.05$), *** ($p \leq 0.001$), **** ($p \leq 0.0001$). Ordinary one-way ANOVA for comparison with vehicle standard. Values are mean ± SEM.

	standard	peptide in blood dilutions							positive control
	vehicle	1:20000	1:10000	1:5000	1:1000	1:500	1:100	1:10	1% Triton X-100
%Hemolysis	0.00	0.20	0.37	0.30	0.13	0.26	0.40	4.43	100.00
SEM	(± 0.07)	(± 0.06)	(± 0.12)	(± 0.08)	(± 0.07)	(± 0.26)	(± 0.12)	(±0.64)	(± 18.17)
P Value		> 0.9999	> 0.9999	> 0.9999	> 0.9999	0.9999	> 0.9999	0.9968	< 0.0001
Summary		ns	ns	ns	ns	ns	ns	ns	****

Supplementary Information References

1 Bhattacharyya, M., Gupta, K., Gowd, K. H. & Balaram, P. Rapid mass spectrometric determination of disulfide connectivity in peptides and proteins. *Molecular BioSystems* **9**, 1340-1350, (2013).
