# Supplementary material for: Enzyme-responsive progelator cyclic peptides for minimally invasive delivery to the heart post-myocardial infarction
Source: Nat Commun. 2019 Apr 15;10:1735. doi: 10.1038/s41467-019-09587-y (PMC6465301; doi:10.1038/s41467-019-09587-y)
Supplement: Supplementary file 2 — Description of Additional Supplementary Files [file 41467_2019_9587_MOESM2_ESM.docx]

Description of Additional Supplementary Files

**Supplementary Movie 1.** Catheter injection of KFDFCyclic progelator. Video shows smooth injection and subsequent gelation upon enzyme activation.

**Supplementary Data 1.** PDB file of KLDL SAP in main text Fig. 2.

**Supplementary Data 2.** PDB file of KFDF SAP in main text Fig. 2.

**Supplementary Data 3.** PDB file of KLDLControl SAP product analogue in main text Fig. 2.

**Supplementary Data 4.** File 4. PDB file of KFDFControl SAP product analogue in main text Fig. 2.

**Supplementary Data 5.** PDB file of KLDLLinear SAP in main text Fig. 2.

**Supplementary Data 6.** PDB file of KFDFLinear SAP in main text Fig. 2
