# Supplementary material for: Enzyme-responsive progelator cyclic peptides for minimally invasive delivery to the heart post-myocardial infarction
Source: Nat Commun. 2019 Apr 15;10:1735. doi: 10.1038/s41467-019-09587-y (PMC6465301; doi:10.1038/s41467-019-09587-y)
Supplement: Supplementary file 10 — Reporting Summary [file 41467_2019_9587_MOESM10_ESM.pdf]

nature research

Corresponding author(s): Karen L. Christman and Nathan C. Gianneschi

Reporting Summary

Nature Research wishes to improve the reproducibility of the work that we publish. This form provides structure for consistency and transparency in reporting. For further information on Nature Research policies, see Authors & Referees and the Editorial Policy Checklist.

Statistical parameters

When statistical analyses are reported, confirm that the following items are present in the relevant location (e.g. figure legend, table legend, main text, or Methods section).

n/a	Confirmed	
☐	☒	The exact sample size (*n*) for each experimental group/condition, given as a discrete number and unit of measurement
☐	☒	An indication of whether measurements were taken from distinct samples or whether the same sample was measured repeatedly
☐	☒	The statistical test(s) used AND whether they are one- or two-sided<br>*Only common tests should be described solely by name; describe more complex techniques in the Methods section.*
☐	☒	A description of all covariates tested
☐	☒	A description of any assumptions or corrections, such as tests of normality and adjustment for multiple comparisons
☐	☒	A full description of the statistics including central tendency (e.g. means) or other basic estimates (e.g. regression coefficient) AND variation (e.g. standard deviation) or associated estimates of uncertainty (e.g. confidence intervals)
☐	☒	For null hypothesis testing, the test statistic (e.g. *F*, *t*, *r*) with confidence intervals, effect sizes, degrees of freedom and *P* value noted<br>*Give P values as exact values whenever suitable.*
☒	☐	For Bayesian analysis, information on the choice of priors and Markov chain Monte Carlo settings
☒	☐	For hierarchical and complex designs, identification of the appropriate level for tests and full reporting of outcomes
☒	☐	Estimates of effect sizes (e.g. Cohen's *d*, Pearson's *r*), indicating how they were calculated
☐	☒	Clearly defined error bars<br>*State explicitly what error bars represent (e.g. SD, SE, CI)*

Our web collection on statistics for biologists may be useful.

Software and code

Policy information about availability of computer code

Data collection	1. FibPredictor was utilized as a commercially available software for generating native-like amyloid fibril structures, as previously published (DOI: 10.1007/s00894-016-3066-1). This software is available online at: http://nanohub.org/resources/fibpredictor<br><br>2. DichroCalc was used to calculate theoretical CD spectra using predicted PDB files from FibPredictor. This web interface, which predicts secondary structure type using a variety of matrix method parameters, is described as previously published (DOI: 10.1093/bioinformatics/btp016). This commercially available software is available at: http://comp.chem.nottingham.ac.uk/dichrocalc/<br><br>3. Disconnect open-source software was used to predict Tandem-MS peptide fragmentation patterns for peptides containing disulfide bonds. This software is described as previously published (DOI: 10.1039/C3MB25534D and DOI: 10.1371/journal.pone.0044913). This software is available at http://www.mmass.org.
Data analysis	GraphPad Prism 6 was used to plot all spectra and perform statistical analysis, as described in the methods section.

For manuscripts utilizing custom algorithms or software that are central to the research but not yet described in published literature, software must be made available to editors/reviewers upon request. We strongly encourage code deposition in a community repository (e.g. GitHub). See the Nature Research guidelines for submitting code & software for further information.

Data

Policy information about availability of data

All manuscripts must include a data availability statement. This statement should provide the following information, where applicable:

- Accession codes, unique identifiers, or web links for publicly available datasets
- A list of figures that have associated raw data
- A description of any restrictions on data availability

The authors declare that all data supporting the findings of this study are available within the article and its supplementary information files.

Field-specific reporting

Please select the best fit for your research. If you are not sure, read the appropriate sections before making your selection.

☒ Life sciences ☐ Behavioural & social sciences

For a reference copy of the document with all sections, see nature.com/authors/policies/ReportingSummary-flat.pdf

Life sciences

Study design

All studies must disclose on these points even when the disclosure is negative.

Sample size	Sample size for test animals with peptide injection was n=5 for this proof-of-concept study.
Data exclusions	No data points were excluded.
Replication	All attempts at replication were successful.
Randomization	Animals were randomly selected to recieve the various treatments. There was no predetermination of treatment.
Blinding	n/a

Materials & experimental systems

Policy information about availability of materials

n/a	Involved in the study
☐	☒ Unique materials
☐	☒ Antibodies
☐	☒ Eukaryotic cell lines
☐	☒ Research animals
☒	☐ Human research participants

Unique materials

Obtaining unique materials	Synthesis of the unique, functionalized cyclic peptides are described in the text. Characterization of these materials is also included.

Antibodies

Antibodies used	Primary Antibodies:<br>Mouse-anti-rat-α-actinin (sarcomeric) antibody (A7811) from Sigma<br>Mouse-anti-rat CD68 (MCA341R) from Biorad<br>Rat-anti-rabbit cleaved caspase-3 (9664) from Cell Signaling<br><br>Secondary Antibodies:<br>Goat-anti-mouse Alexa Fluor 488 (A-11001) from Thermo Fisher<br>Goat-anti-rabbit 647 (A-31573) from Thermo Fisher
Validation	The validation results for all commercial antibodies used are available on the manufacturers website.

Eukaryotic cell lines

Policy information about cell lines

Cell line source(s)	Unspun whole human blood (citrated, non-heparinized) from Biological Specialty Corporation (LS23 95099) were used within

Cell line source(s)	20 days of receipt.<br><br>Human fetal cardiomyocyte progenitor cells (hCMPCs) were isolated from human fetal hearts as described in the methods section.
Authentication	Whole human blood was tested in accordance with AABB guidelines and FDA requirements for infectious disease state tests. Tests were negative for Hepatitis B surface antigen, HVB NAT, HIV 1&2 antibody, HIV NAT, HCV antibody, HCV NAT, syphilis, west nile virus NAT, HTLV I/II (leukocyte products only), and T.cruzi antibodies.
Mycoplasma contamination	Stem cells were confirmed mycoplasma negative before undertaking any experiments.
Commonly misidentified lines<br>(See ICLAC register)	n/a

Research animals

Policy information about studies involving animals; ARRIVE guidelines recommended for reporting animal research

Animals/animal-derived materials	Female Sprague-Dawley rats

Method-specific reporting

n/a	Involved in the study
☒	☐ ChIP-seq
☒	☐ Flow cytometry
☒	☐ Magnetic resonance imaging
